# The epithelial splicing regulator *ESRP2* is epigenetically repressed by DNA hypermethylation in Wilms tumour and acts as a tumour suppressor

**DOI:** 10.1002/1878-0261.13101

**Published:** 2021-09-28

**Authors:** Danny Legge, Ling Li, Whei Moriarty, David Lee, Marianna Szemes, Asef Zahed, Leonidas Panousopoulos, Wan Yun Chung, Yara Aghabi, Jasmin Barratt, Richard Williams, Kathy Pritchard‐Jones, Karim T.A. Malik, Sebastian Oltean, Keith W. Brown

**Affiliations:** ^1^ School of Cellular and Molecular Medicine University of Bristol UK; ^2^ Institute of Biomedical & Clinical Sciences University of Exeter Medical School UK; ^3^ Cancer Section UCL Great Ormond Street Institute of Child Health London UK

**Keywords:** DNA methylation, epigenetics, ESRP2, MET, Wilms tumour

## Abstract

Wilms tumour (WT), an embryonal kidney cancer, has been extensively characterised for genetic and epigenetic alterations, but a proportion of WTs still lack identifiable abnormalities. To uncover DNA methylation changes critical for WT pathogenesis, we compared the epigenome of foetal kidney with two WT cell lines, filtering our results to remove common cancer‐associated epigenetic changes and to enrich for genes involved in early kidney development. This identified four hypermethylated genes, of which *ESRP2* (epithelial splicing regulatory protein 2) was the most promising for further study. *ESRP2* was commonly repressed by DNA methylation in WT, and this occurred early in WT development (in nephrogenic rests). *ESRP2* expression was reactivated by DNA methyltransferase inhibition in WT cell lines. When *ESRP2* was overexpressed in WT cell lines, it inhibited cellular proliferation *in vitro*, and *in vivo* it suppressed tumour growth of orthotopic xenografts in nude mice. RNA‐seq of the ESRP2‐expressing WT cell lines identified several novel splicing targets. We propose a model in which epigenetic inactivation of *ESRP2* disrupts the mesenchymal to epithelial transition in early kidney development to generate WT.

## Graphical abstract

*ESRP2* regulates alternative splicing events that are critical for the mesenchymal to epithelial transition (MET) that occurs during kidney development. We show that in Wilms tumour, *ESRP2* is commonly inactivated by DNA methylation at an early stage of carcinogenesis. We propose that epigenetic inactivation of *ESRP2* disrupts the regulation of alternative splicing during MET. Disrupted MET leads in turn to persistence of undifferentiated foetal kidney cells that may progress to a tumour.

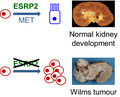

**Abbreviations**
ANOVA: analysis of variance
Aza: 5‐aza‐2’‐deoxycytidine
BCH: Bristol Children’s Hospital
DMEM: Dulbecco’s modified Eagle’s medium
Dox: doxycycline
FK: foetal kidney
MCIP: methyl CpG immunoprecipitation
MET: mesenchymal–epithelial transition
NK: normal kidney
NR: nephrogenic rest
NT: normal tissue
qPCR: quantitative real‐time PCR
RMH: Royal Marsden Hospital
RNA‐seq: RNA sequencing
WT: Wilms tumour

## 1 Introduction

Wilms tumour (WT; nephroblastoma) is an embryonal kidney cancer [1, 2], which originates from foetal kidney (FK), due to the failure of the mesenchymal to epithelial transition (MET) that the metanephric blastema undergoes during early nephrogenesis. Premalignant lesions (nephrogenic rests; NRs) are often found as microscopic lesions in the normal kidney (NK) adjacent to WTs [3]. It is hypothesised that genetic and epigenetic defects occur during renal development that block MET, leading to the formation of NRs, some of which progress to WT [1, 2, 3].

The molecular events underlying WT pathogenesis involve an array of genetic and epigenetic defects [2]. The earliest genetic mutations in WT were found in the *WT1* gene, which plays a critical role in regulating MET during nephrogenesis [2]. The Wnt pathway is also vital in renal development [2], and mutations in Wnt pathway components including *CTNNB1* [4, 5] and *WTX* (*AMER1*) [6] have also been found in WT. Recent genome‐wide sequencing studies have identified mutations in microRNA‐processing genes, such as *DROSHA*, *DICER* and *DGCR8*, and mutations in other renal developmental regulators, including *SIX1*, *SIX2* and *SALL1* [7, 8, 9, 10, 11, 12]. Most of these events show no strong association with clinical outcome, but *TP53* mutations are found in the rare anaplastic variant of WT, which has a much poorer prognosis than other subtypes [13].

Epigenetic alterations are also common in WT, especially at the 11p15 locus, where the frequent loss of imprinting of the foetal growth factor gene *IGF2* is associated with DNA hypermethylation at *H19* [14]. Other epigenetic alterations in WT include loss of imprinting at 11p13 involving imprinted *WT1* transcripts [14, 15], global hypomethylation [16], DNA hypermethylation at individual tumour suppressor genes such as *RASSF1A* [16, 17, 18], and long‐range epigenetic silencing of the *PCDHG@* gene clusters [19].

Despite the identification of many loci with genetic and/or epigenetic lesions in WT, a proportion of WTs still lack identifiable driver defects, implying that additional novel genes are involved in WT pathogenesis [7]. We previously used genome‐wide DNA methylation analysis to identify novel epigenetic lesions in WT [19], and here, we report further studies comparing WT cell lines to foetal kidney. We have identified novel differentially methylated genes, one of which is the alternative splicing regulator *ESRP2* (epithelial splicing regulatory protein 2). *ESRP2* is known to be important in epithelial to mesenchymal transitions and MET [20], suggesting that epigenetic deregulation of MET may be an important factor in WT development. We show that *ESRP2* is frequently silenced by DNA hypermethylation in WT and that it acts as a tumour suppressor gene, regulating alternative splicing in novel genes, some of which affect pathways known to be important in kidney development.

## 2 Materials and methods

### 2.1 Ethical statement

WT samples were from Bristol Children’s hospital (BCH), or from collaborators at the Royal Marsden Hospital (RMH), as part of a UK collaboration. Samples were obtained with informed written consent (from parent and/or legal guardian for children less than 18 years old) and with appropriate ethical approval (E5797, Southwest – Central Bristol Research Ethics Committee (UK)). All methods were performed in accordance with the relevant regulations specified in the UK Human Tissue Act 2004. The study methodologies conformed to the standards set by the Declaration of Helsinki. All animal experiments and procedures were approved by the UK Home Office in accordance with the Animals (Scientific Procedures) Act 1986. Mice were maintained at the Biological Services Unit, University of Exeter, UK. Housing and handling of mice have been done according to the UK Home Office Code of Practice: https://assets.publishing.service.gov.uk/government/uploads/system/uploads/attachment_data/file/388895/COPAnimalsFullPrint.pdf.

### 2.2 Cell lines

WT cell lines Wit49 (a kind gift from Professor Herman Yeger, University of Toronto) [21] and 17.94 (established in our own laboratory; available from DSMZ (German Collection of Microorganisms and Cell Cultures), https://www.dsmz.de/dsmz) [22] were grown in Dulbecco’s modified Eagle’s medium (DMEM) with 10% FBS, 100 U·mL^−1^ penicillin, 0.1 mg·mL^−1^ streptomycin, and 2 mm l‐glutamine, at 37 °C in 5% CO_2_. WT cell line identity was confirmed by short tandem repeat analysis (Fig. S1).

The V200 and E200L cell lines were derived by transfecting Wit49 cells with the inducible expression vector pBIG2r [23], either empty (V200) or containing an *ESRP2* cDNA insert (E200L). *ESRP2* cDNA was amplified by PCR from IMAGE clone 4810948, using a forward primer containing a BamHI site and a reverse primer containing an EcoRV site plus a FLAG tag (Table S1), then ligated into BamHI/EcoRV‐digested pBIG2r (Fig. S2A). Transfected cells were selected and maintained in 50 µg·mL^−1^ hygromycin B (Santa Cruz Biotechnology). Only the *ESRP2*‐transfected Wit49 cells (E200L) expressed vector‐derived *ESRP2* RNA (Fig. S2B). *ESRP2* expression was induced with 2‐5 µg·mL^−1^ doxycycline (Dox, Sigma), with maximum ESRP2 protein expression at 72 to 96 h postinduction (Fig. S2C).

### 2.3 Transient transfection

WT cell lines Wit49 and 17.94 were seeded into 6‐well plates (2 × 10^5^ cells·well^−1^) and transfected with 1 µg plasmid expressing FLAG‐tagged *Esrp1* or *Esrp2*, or empty vector (pIBX‐C‐FF‐B‐Esrp1/2 [24]), using FuGENE 6 (Promega), according to the manufacturer’s instructions. Transfected cells were selected with 2.5 µg·mL^−1^ blasticidin (Sigma) after three days, and after 5 days, adherent cells were trypsinised and counted.

### 2.4 Cell growth assays

For mass culture assays, cells were seeded into 6‐well plates (1 × 10^6^ cells·well^−1^) and treated with 2 µg·mL^−1^ doxycycline or DMSO vehicle control, with medium changes every 3 days. Cells were trypsinised and counted using a Countess Cell Counter and trypan blue stain to exclude dead cells.

For colony assays, cells were seeded into 6‐well plates (2 × 10^5^ cells·well^−1^) and treated with 2 µg·mL^−1^ doxycycline or DMSO vehicle control. Medium was changed every 3 days, and then at 14 days, cells were fixed, stained with methylene blue and colonies counted manually.

To monitor proliferation in real time, cells were seeded in 24‐well plates (5 × 10^3^ cells·well^−1^) and images were taken in four different fields per well every 2 h (IncuCyte ZOOM live cell imaging system; Essen BioScience) and phase confluence was calculated as a surrogate for growth.

### 2.5 Transwell assay

Cells were pretreated for 4 days with 2 µg·mL^−1^ doxycycline or control media and then seeded into Transwell inserts (2 × 10^5^ cells·insert^−1^ in FBS‐free DMEM; 8 µm pore, PET membrane; Falcon, 353093) in a 6‐well plate. Wells were filled with 1.7 mL 10% FBS in DMEM to produce a chemotactic gradient. After 24 h, inserts were washed and cells on underside of membrane were fixed and stained with crystal violet and counted manually using light microscopy.

### 2.6 Scratch assay

Cells were seeded into 24‐well plates (7.5 × 10^5^ cells·well^−1^) and treated with 2 µg·mL^−1^ doxycycline or DMSO vehicle control, prior to a scratch being performed manually in the centre of each well. Wells were washed with PBS to remove dead cells, control/doxycycline media replaced, and wells were analysed at 24 and 48 h via Widefield microscopy, using ImageJ software to determine percentage wound closure.

### 2.7 Cell Trace Violet (CTV) proliferation assay

1x10^6^ cells were incubated for 20 min at 37 °C in the dark in 1 mL of diluted CTV stain (Thermo Fisher; C34571; prepared according to manufacturer’s instructions), then staining was quenched using 10% FBS in DMEM, and cells were seeded into T12.5 flasks. Controls were made by fixing 3x10^5^ stained cells in 1% paraformaldehyde and stored at 4 °C in the dark. Seeded cells in T12.5 flasks were treated for 6 days with 2 µg·mL^−1^ doxycycline or control media, and intensity of CTV staining was analysed using a NovoCyte Flow Cytometer and flowjo software.

### 2.8 5‐Aza‐2’‐deoxycytidine treatment

Cells were incubated in medium containing 2 µm 5‐aza‐2’‐deoxycytidine (Aza; Sigma, Gillingham, UK) or drug solvent (DMSO) for up to 6 days, with a medium change every 2 days.

### 2.9 Xenografting into nude mice

V200 and E200L cells were transduced with lentivirus expressing firefly luciferase (Amsbio LVP326), and transduced cells were selected with blasticidin, according to the manufacturer’s protocol. For orthotopic kidney implantation, male nude mice (2 months old; Charles River) were anaesthetised using isoflurane, an incision was performed in the left flank of the mice, the kidney was exteriorised and 3 × 10^6^ cells were injected. Mice were imaged twice weekly (Xenogen IVIS), following intraperitoneal injection with luciferin. When a bioluminescent signal above background was detected (demonstrating the establishment of tumour growth), mice were injected intraperitoneally with doxycycline three times/week (50 mg·kg^−1^ in 5% glucose). Mice were culled either when tumours grew to the maximum allowed size (10mm in diameter, according to the animal licence) or after two months of imaging. The sample size was determined by power calculations using existing data from similar experiments performed routinely in Dr Oltean’s lab. More specifically, the sample size was obtained to be able to see a significant difference (*P* > 0.05) for tumour growth with a power value of 0.80 (> 80%). We have used statistical principles and formulas available on the following websites: www.nc3rs.org.uk; http://www.statisticalsolutions.net/pss_calc.php. We have not done randomisation in the animal experiments, and there was no blinding of the investigator.

### 2.10 DNA extraction and methyl CpG immunoprecipitation (MCIP)

DNA was extracted from WT cell lines with a DNeasy kit (Qiagen, Manchester, UK). Human foetal kidney DNA was obtained from BioChain. MCIP was performed as described previously [25] by co‐hybridising methylation‐enriched DNA fractions with input DNA onto a custom microarray (NimbleGen), based on design 2006‐04‐28_HG18_Refseq_Promoter (see GEO entry for further details). Statistical analyses by ChIPMonk software (https://www.bioinformatics.babraham.ac.uk/projects/chipmonk/) used windowed *t*‐tests to identify differentially methylated genes (Table S2). MCIP data are accessible through GEO Series accession number GSE153047: https://www.ncbi.nlm.nih.gov/geo/query/acc.cgi?acc=GSE153047.

### 2.11 Pyrosequencing

DNA was purified by phenol/chloroform extraction, bisulphite converted (EZ DNA Methylation Gold kit; Zymo Research), amplified using a PyroMark PCR kit (Qiagen) and pyrosequenced on a PyroMark Q96 instrument (Qiagen), using primers listed in Table S3.

### 2.12 RNA extraction, cDNA synthesis and RT‐PCR

Total RNA was extracted using TriReagent (Sigma) and DNase treated with TURBO DNA‐free (Ambion, Gloucester, UK). Human foetal kidney RNA was obtained from BioChain. cDNA was synthesised using the Superscript IV RT‐PCR system (Invitrogen, Gloucester, UK). Gene‐specific primers (Table S1) were used for end‐point PCR (HotStarTaq Plus DNA Polymerase; Qiagen), to detect inclusion or exclusion of alternative exons, after electrophoresis on agarose gels (1.5%). Quantitative real‐time PCR (qPCR) using gene‐specific primers (Table S1) was performed using QuantiNova SYBR Green Mix (Qiagen) on an MX3000P real‐time PCR machine (Stratagene), normalising the amount of target gene to the endogenous level of *TBP*. Human universal RNA (Agilent) was used as a reference to standardise results between qPCR batches.

### 2.13 Protein extraction and western blotting

Cells were washed with ice‐cold PBS and lysed in cell lysis buffer (Cell Signaling, London, UK), with complete mini‐inhibitors (Roche) for 10 min on ice, and then sonicated for 5 min (Diagenode, Bioruptor). 25 µg proteins were separated on SDS/polyacrylamide gels and analysed by western blotting. Primary antibodies were against ESRP2 (rabbit, Abcam ab155227), FLAG (mouse, Sigma F3165) and β‐ACTIN (rabbit, Abcam AB8227), followed by secondary HRP‐labelled anti‐rabbit IgG (Sigma A6154) or anti‐mouse IgG (Sigma A9044). Chemiluminescence detection was with Lumiglo (KPL).

### 2.14 Immunofluorescence

Cells were grown on sterile glass slides, fixed for 30 min at room temperature in 1% paraformaldehyde in PBS, permeabilised for 10 min in 0.5% Triton X‐100 in PBS and finally rinsed in 50mM glycine in PBS. Fixed cells were stained using a primary antibody against FLAG (mouse, Sigma F3165) and secondary antibody against mouse IgG (Alexa Fluor 488‐labelled; Invitrogen) to detect transfected ESRP2, together with Alexa Fluor 594‐labelled phalloidin (Invitrogen) to detect actin. Antibodies were diluted in PBS + 1% bovine serum albumin, containing 0.1 µg·mL^−1^ DAPI to image nuclei. Slides were mounted in Fluoroshield (Sigma) and examined with a confocal microscope, acquiring eight images at 1 µm spacing/field. Maximum intensity projections were merged using ImageJ software (http://imagej.nih.gov/ij/).

### 2.15 RNA sequencing (RNA‐seq)

RNA was extracted from E200L cells 96 h after treatment with 2 µg·mL^−1^ doxycycline, or control solvent (DMSO), using an RNAeasy kit (Qiagen), then DNase treated, and quality confirmed using an Agilent ScreenTape RNA assay. Two biological replicates were used for RNA‐seq (i.e. four samples total). Sequencing libraries were prepared from total RNA (500 ng) using the TruSeq Stranded mRNA Library Preparation Kit (Illumina, Inc., Cambridge, UK) and uniquely barcoded adapters (RNA LT adapters, Illumina, Inc). Libraries were pooled equimolarly for sequencing, which was carried out on the NextSeq500 instrument (Illumina, Inc.) using the NextSeq High Output v2 150‐cycle kit (Illumina, Inc.). Approximately 300 million paired reads (passing filter, PF) were obtained, divided between the four experimental samples. NextSeq Control Software version 2.0.0 and RTA v2.4.6 were used for instrument control and primary analysis, respectively. Reads from the four samples were mapped to the human genome (hg19) using the new Tuxedo Suite of programs (HISAT2, StringTie, Ballgown; https://www.ncbi.nlm.nih.gov/pubmed/?term=27560171). To identify RNA splicing alterations, the four BAM files generated by HISAT2 were used as input for rMATS ([26] http://rnaseq‐mats.sourceforge.net/user_guide.htm). Bam files were viewed in the Integrative Genomics Viewer (http://software.broadinstitute.org/software/igv/) to produce Sashimi plots of alternative splicing. RNA‐seq data are accessible through GEO Series accession number GSE154496: https://www.ncbi.nlm.nih.gov/geo/query/acc.cgi?acc=GSE154496.

### 2.16 Statistical analysis

Comparisons of two datasets were performed using Student’s *t*‐test or a Mann–Whitney *U*‐test, depending on whether the data met the normal distribution. A comparison of three or more groups was performed using one‐way analysis of variance (ANOVA) with Dunnett’s post‐test or using Tukey’s pairwise test. The Chipmonk software used for MCIP analysis and the rMATS software used for RNA‐seq analysis use Benjamini and Hochberg FDR correction for multiple testing. For smaller numbers of samples, Bonferroni correction was used for multiple testing. Numbers of samples quoted in figure legends (*n*) refer to biological replicates. *P* < 0.05 was considered to indicate a statistically significant difference.

## 3 Results

### 3.1 Genome‐wide DNA methylation analysis

**Fig. 1:**
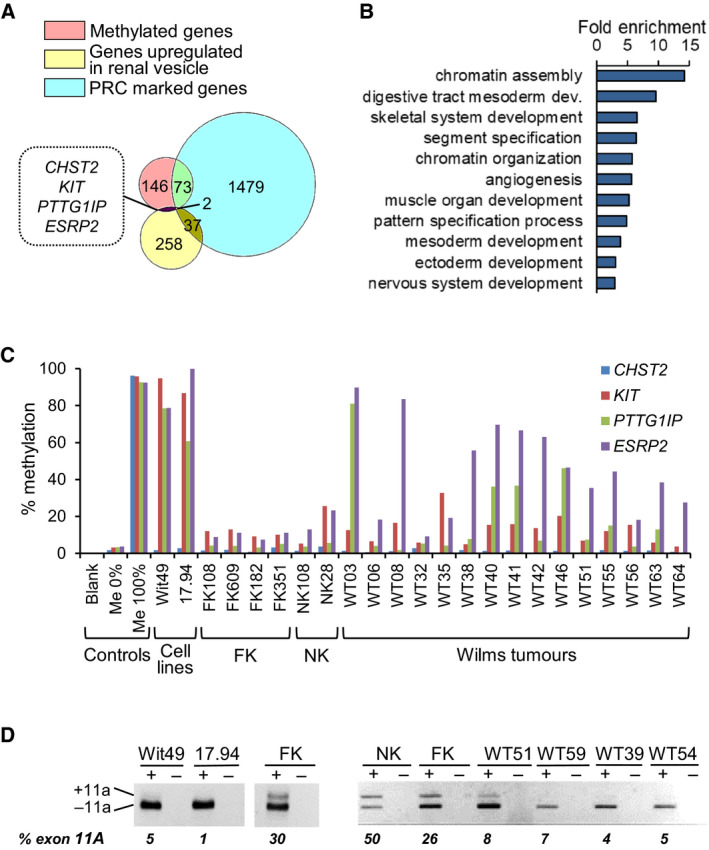
Identification of *ESRP2* as a candidate hypermethylated gene. (A) Venn diagram showing filtering of the 225 methylated genes that were identified by methyl CpG immunoprecipitation (MCIP), firstly by negative selection for genes that are polycomb repressive complex (PRC) marked in embryonic stem cells and secondly by positive selection for genes that are upregulated in the renal vesicle during kidney development. The full list of methylated genes and filtered lists are shown in Table S2. dev., development. (B) Gene Ontology analysis of the 225 methylated genes. Only categories with a fold enrichment > 3 are shown; Table S4 for full results. (C) Bar chart of *CHST2*, *KIT*, *PTTG1IP* and *ESRP2* DNA methylation. Controls (Blank, Me 0% (unmethylated DNA control), Me 100% (fully methylated DNA control)), Cell lines (Wilms tumour cell lines, *n* = 2), FK (foetal kidney, *n* = 4), NK (normal kidney, *n* = 2) and Wilms tumours (*n* = 15). DNA methylation was assayed by pyrosequencing; Table S3 for pyrosequencing primers. (D) Alternative splicing of *ENAH* exon 11A was analysed by RT‐PCR followed by agarose gel electrophoresis in 2 WT cell lines (Wit49 and 17.94), FK, NK and 4 WTs. Representative of *n* = 3.

We used MCIP to identify 225 genes that were hypermethylated in two WT cell lines compared with foetal kidney (Fig. 1A and Table S2). Gene ontology analysis showed that these genes were particularly involved in chromatin organisation, developmental processes and transcriptional regulation (Fig. 1B and Table S4). To distinguish genes that were methylated specifically in WT, two filters were applied: (a) genes were removed that are polycomb repressive complex marked in embryonic stem cells, since such genes are predisposed to DNA methylation in a wide range of human cancers [27, 28, 29] and therefore might not be WT‐specific; (b) positive selection was applied for genes that are upregulated in early nephrogenesis, since their inactivation could induce the MET block that is critical for WT development [2]. Using these criteria, four candidate genes were pinpointed: *CHST2*, *KIT*, *PTTG1IP* and *ESRP2* (also known as *RBM35B*) (Fig. 1A and Table S2), of which *ESRP2* was the most consistently methylated in WT (Fig. 1C).

*ESRP2* was particularly attractive for further study, because of its known involvement in epithelial to mesenchymal transitions in cancer [30]. Support for a role in WT came from examination of the ESRP2 target *ENAH*. ESRP2 induces inclusion of the epithelial‐specific exon 11a in *ENAH* RNA transcripts [24]. Using RT‐PCR, less exon 11A was found expressed in WTs compared with normal kidney (NK) and foetal kidney (FK), consistent with downregulation of *ESRP2* in WT (Fig. 1D). We therefore went onto examine DNA methylation and expression of *ESRP2* in two large cohorts of WTs using pyrosequencing (Figs. S3 and S4).

### 3.2 DNA methylation of ESRP2 in Wilms tumour

**Fig. 2:**
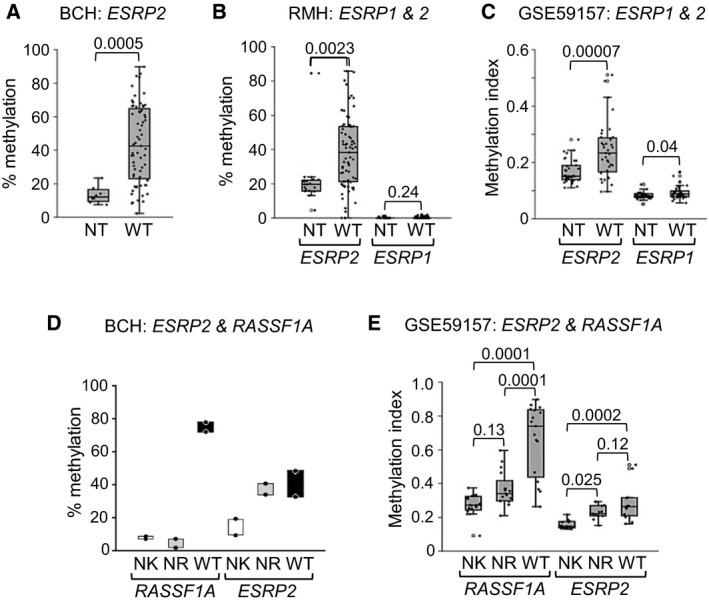
*ESRP2* is hypermethylated in Wilms tumours and nephrogenic rests. (A) Dot‐boxplot of *ESRP2* DNA methylation in the BCH cohort. NT (normal tissue) *n* = 8 (4 NK (normal kidney) and 4 FK (foetal kidney), WT (Wilms tumour) *n* = 65, *P* value from *t*‐test. (B) Dot‐boxplot of *ESRP2* and *ESRP1* DNA methylation in the RMH cohort. *ESRP2*: NT *n* = 18 (all NK), WT *n* = 73; *ESRP1*: NT *n* = 15 (all NK), WT *n* = 69, *P* values from *t*‐test. (C) Dot‐boxplot of *ESRP2* and *ESRP1* DNA methylation in dataset GSE59157. NT *n* = 36 (all NK), WT *n* = 37, *P* values from *t*‐test. (D) *RASSF1A* and *ESRP2* methylation in nephrogenic rests in the BCH cohort. Two sets of matched NK, NR (nephrogenic rest) and WT are shown. (E) Dot‐boxplot of *RASSF1A* and *ESRP2* methylation in nephrogenic rests in the GSE59157 data set. 17 sets of matched NK, NR and WT are shown for *RASSF1A* methylation and 13 sets for *ESRP2* methylation, from individuals where the WT was hypermethylated compared with the matched NK. *P* values from Tukey’s pairwise test. DNA methylation was assayed by pyrosequencing in A to D and by Illumina Human Methylation 450 bead arrays in E.

The first cohort of WTs from Bristol Children’s Hospital (BCH) consisted of tumour samples of all stages, obtained at surgical resection, prechemotherapy. 72% of these WTs were hypermethylated at the *ESRP2* (DNA methylation > 25%) compared with normal tissue (NT) (Fig. 2A and Fig. S4A). The second cohort from the Royal Marsden Hospital (RMH) were from stages 1 to 3, taken at surgical resection, postchemotherapy. In this different cohort, 78% of WTs were hypermethylated (Fig. 2B and Fig. S4C). *ESRP1* DNA methylation was also tested in the RMH cohort and found to be very low (< 2%) in NT and WT, and not significantly different (Fig. 2B). Additional independent DNA methylation data were extracted for the *ESRP1* and *ESRP2* promoters from the data set GSE59157, which showed hypermethylation of *ESRP2* in WTs, with much lower methylation of *ESRP1*, that was only marginally different between NT and WT (Fig. 2C). Thus, *ESRP2* DNA was hypermethylated in three independent cohorts of WTs, but the *ESRP2* paralog *ESRP1* was not hypermethylated.

There was no significant association between tumour stage and *ESRP2* DNA methylation (Fig. S5A, B), nor between *ESRP2* methylation and survival (Fig. S6), nor between tumour histology and *ESRP2* methylation (Fig. S7A, C).

*ESRP2* is located on chromosome 16q22, a chromosomal region showing frequent loss of heterozygosity (LOH) in WT [31]. No difference was observed in the *ESRP2* methylation in WTs with or without 16q LOH (Fig. S5C).

Most WTs are thought to develop via premalignant lesions (NRs) [3]. To characterise the phase of WT development at which *ESRP2* DNA methylation occurs, it was assayed in two sets of matched NK, NR and WT. *ESRP2* was found to be at a similar level of hypermethylation in NRs and matched WTs compared with NKs (Fig. 2D). In contrast, *RASSF1A*, a tumour suppressor gene frequently hypermethylated in WT [18], was not hypermethylated in NRs (Fig. 2D), as previously reported [16]. Methylation values were also extracted for NRs from data set GSE59157, and similarly, *ESRP2* was significantly more methylated in both NR and WT compared with NK (Fig. 2E), but *RASSF1A* was only hypermethylated in WTs and not NRs compared with NK (Fig. 2E).

To investigate whether epigenetic changes, including *ESRP2* hypermethylation, are associated with other clinical and molecular features, the BCH cohort of WTs were grouped by hierarchical clustering of DNA methylation values at four loci: *ESRP2*, the *WT1* antisense regulatory region [15], *H19* [14] and *RASSF1A* [18] (Fig. S8 and Table S5). Interestingly, of the 22 WTs studied for *WT1* mutations, all six *WT1*‐mutant WTs were in the same cluster (group 3), whereas ten of the *WT1* wild‐type WTs were in group 1 or 2 and six in group 3 (*P* = 0.015, Fisher exact test). This difference in epigenetic profiles between *WT1*‐mutant and wild‐type WTs is supported by similar findings in a recent comprehensive characterisation of molecular defects in WT [7].

*ESRP2* DNA hypermethylation was also observed in 10 of 16 (63%) non‐WT childhood renal tumours (Fig. S9A), especially in clear cell sarcomas of the kidney and in rhabdoid tumours. In data sets GSE73187 and GSE4487, *ESRP2* was also found to be hypermethylated in clear cell sarcomas (Figs S9B, C) and rhabdoid tumours (Fig. S9C). Interestingly, examination of TCGA data showed DNA methylation changes in *ESRP2* in several adult cancers, including hypermethylation in two adult kidney cancers (renal clear cell carcinoma and renal papillary cell carcinoma; Fig. S9D). This suggests that epigenetic inactivation of *ESRP2* may be involved in the pathogenesis of several types of renal tumours in adults and children, not just in WT.

### 3.3 Expression of *ESRP2* in Wilms tumour

**Fig. 3:**
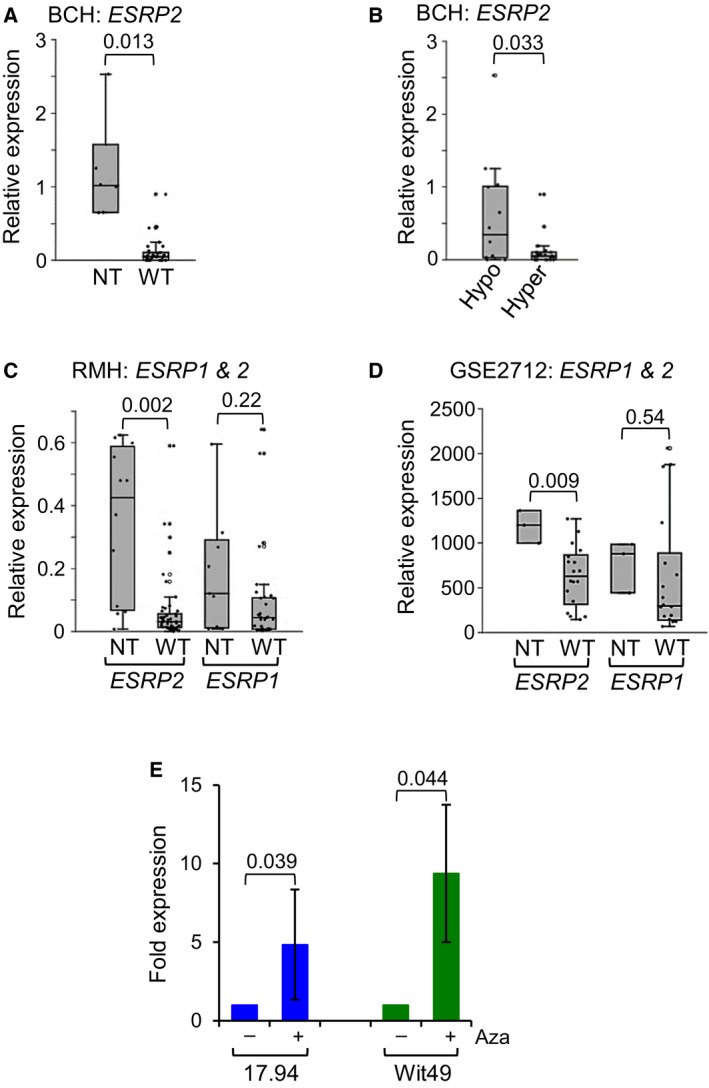
*ESRP2* expression is repressed in Wilms tumour and regulated by DNA methylation. (A) Dot‐boxplot of *ESRP2* RNA expression relative to FK (foetal kidney) in the BCH cohort. NT (normal tissue) *n* = 6 (3 NK (normal kidney) and 3 FK), WT (Wilms tumour) *n* = 32, *P* value from *t*‐test. (B) Dot‐boxplot of *ESRP2* expression in hypomethylated versus hypermethylated samples in the BCH. Hypomethylated *n* = 14 (ESRP2 methylation< 25%, Hypo), hypermethylated *n* = 24 (ESRP2 methylation > 25%, hyper), *P* value from *t*‐test. (C) Dot‐boxplot of *ESRP2* and *ESRP1* RNA expression relative to NT in the RMH cohort. *ESRP2*: NT *n* = 12 (all NK), WT *n* = 51; *ESRP1*: NT *n* = 9 (all NK), WT *n* = 33, *P* values from *t*‐tests. (D) Dot‐boxplot of *ESRP2* and *ESRP1* RNA expression in the GSE2712 data set. NT *n* = 3 (all FK), WT *n* = 18, *P* values from *t*‐tests. (E) 17.94 and Wit49 WT cell lines were treated with 2 µm Aza (5‐aza‐2’‐deoxycytidine) for six days. *ESRP2* RNA levels expressed relative to untreated cells. Results are mean ± SD of *n* = 3, *P* values from paired *t*‐tests. RNA expression was measured by real‐time qPCR, normalised to endogenous levels of *TBP* in A, B, C and E, and by Affymetrix Human Genome U133A arrays in D.

In the BCH cohort, expression of *ESRP2* in WT was very low compared with NT (Fig. 3A and Fig. S4B) and hypermethylation was associated with reduced expression of *ESRP2* (Fig. 3B). In the RMH cohort, the expression of *ESRP2* was also reduced in WT compared with NT (Fig. 3C and Fig. S4D), but *ESRP1* expression was not significantly different (Fig. 3C). In data set GSE2712, *ESRP2* expression was lower in WT compared with NT, but *ESRP1* expression did not differ significantly (Fig. 3D). Like methylation results, there was no relationship between *ESRP2* expression and tumour histology (Fig. S7B, D, E). These results showed that *ESRP2* but not *ESRP1* expression was reduced in WTs compared with NT and that reduced expression of *ESRP2* was associated with hypermethylation. When the two WT cell lines were treated with the DNA methylation inhibitor 5‐aza‐2’‐deoxycytidine (Aza), there was a 5‐ to 10‐fold increase in *ESRP2* RNA expression (Fig. 3E), suggesting a mechanistic link between *ESRP2* methylation and gene expression.

### 3.4 Biological function of *ESRP2* in vitro

**Fig. 4:**
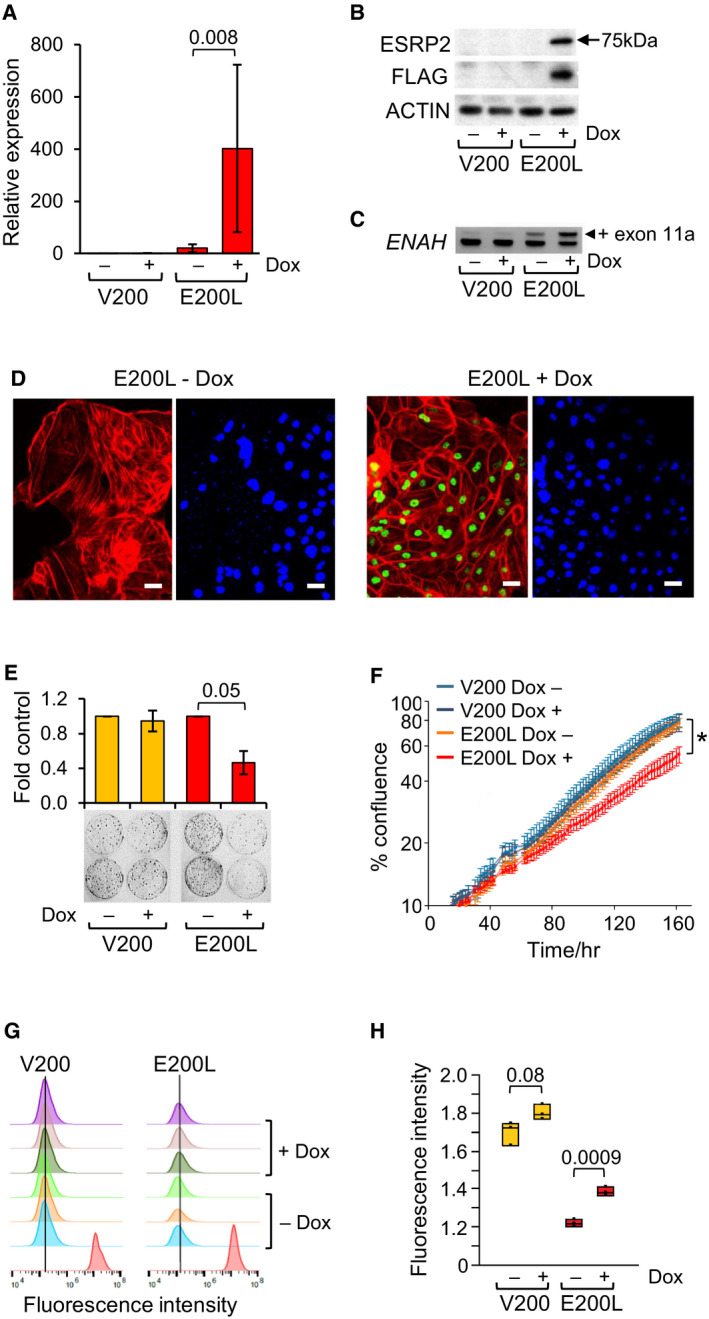
Inducible expression of *ESRP2* in a Wilms tumour cell line. V200 is a control WT cell line (transfected with empty vector), and E200L is a WT cell line expressing doxycycline‐inducible *ESRP2* (Materials and Methods). (A) *ESRP2* RNA expression assayed by qPCR, normalised to endogenous levels of *TBP*, in V200 and E200L cells after 72‐h doxycycline (Dox) induction, shown as fold induction relative to uninduced V200 cells. Results are mean ±SD of *n* = 3, *P* value from paired *t*‐test. (B) ESRP2 protein assayed by western blotting in V200 and E200L cells after 72‐h doxycycline induction. Anti‐ESRP2 detected total ESRP2 protein, anti‐FLAG detected vector‐derived ESRP2 and anti‐ACTIN was used as a loading control. Representative of *n* = 3. (C) Alternative splicing of *ENAH* exon 11A was analysed by RT‐PCR followed by agarose gel electrophoresis to detect different sized amplicons, in V200 and E200L cells after 72‐h doxycycline induction. Representative of *n* = 3. (D) Immunofluorescence of E200L cells, stained for FLAG‐tagged ESRP2 (green) and ACTIN (red) in the left‐hand panels, and for nuclear DNA with DAPI (blue) in the right‐hand panels, after 72‐h doxycycline induction (+Dox), or uninduced (‐Dox). Scale bars = 50 µm. (E) Colony‐forming assay of induced (doxycycline‐treated) and uninduced V200 and E200L cells, shown as fold colony numbers compared to uninduced controls after 14 days. Results are mean ± SD of *n* = 3, *P* values from paired *t*‐test. (F) Cell confluence assay (by IncuCyte), showing growth of induced (doxycycline‐treated) and uninduced V200 and E200L cells. Results are mean ± SD of *n* = 6, *P* value at 162 h from paired *t*‐test. Representative of *n* = 3. (G) H: Cell trace violet (CTV) proliferation assay of induced (doxycycline‐treated) and uninduced V200 and E200L cells. (G) CTV staining of triplicates of induced and uninduced cells showing median fluorescence intensity histograms at 6 days of treatment. Red peaks are controls representing staining of cells at day zero. (H) Dot‐boxplot of quantitation of staining at 6 days (*n* = 3). *P* values from paired *t*‐tests of log‐transformed values.

The results described above suggested that *ESRP2* may have an important functional role in the development of WT. To carry out functional analyses, we initially used transient transfection to constitutively overexpress *ESRP* genes in WT cell lines 17.94 and Wit49. Overexpression of *ESRP2*, but not of *ESRP1*, produced strong growth inhibition in both cell lines (Fig. S10). Due to the strong growth inhibition by *ESRP2*, we were unable to establish stable cell lines using these constitutively active expression vectors. We therefore transfected the WT cell lines with an inducible *ESRP2* expression vector (Fig. S2). Unfortunately, we were unable to establish a stable cell line from 17.94, but the WT cell line Wit49 was successfully transfected, producing the E200L cell line (V200 was the control cell line transfected with empty vector). E200L showed strong doxycycline‐induced expression of *ESRP2* RNA (Fig. 4A) and protein (Fig. 4B), with the expected nuclear localisation of ESRP2 protein (Fig. 4D). Induction of ESRP2 drove the splicing of the known target gene *ENAH* [24] towards its epithelial splice form (+exon 11a; Fig. 4C), demonstrating that the construct produced biologically active ESRP2 in a WT cell line. There was slight leakiness from the expression vector, with more *ESRP2* RNA detected in uninduced E200L cells compared with V200 cells (Fig. 4A), which probably explains the increased level of *ENAH* exon 11a in uninduced E200L cells compared with V200 cells (Fig. 4C).

Overexpression of ESRP2 was associated with an apparent redistribution of actin filaments towards the cell periphery (Fig. 4D), compared to a more cytoplasmic distribution of actin stress fibres in uninduced cells (Fig. 4D), as reported in other systems [32, 33].

ESRP2 overexpression caused decreased colony‐forming efficiency (Fig. 4E), as well as reduced growth rate in mass cultures (Fig. S11). Real‐time analysis of cell density showed a slower cell proliferation rate in the doxycycline‐induced E200L cells (Fig. 4F), associated with a small but significant decrease in the rate of cell division (Fig. 4G, H). Cell invasion (Fig. S12A) and cell motility (Fig. S12B) showed no changes upon induction of ESRP2 expression.

### 3.5 Xenograft assays of *ESRP2* function *in vivo*

**Fig. 5:**
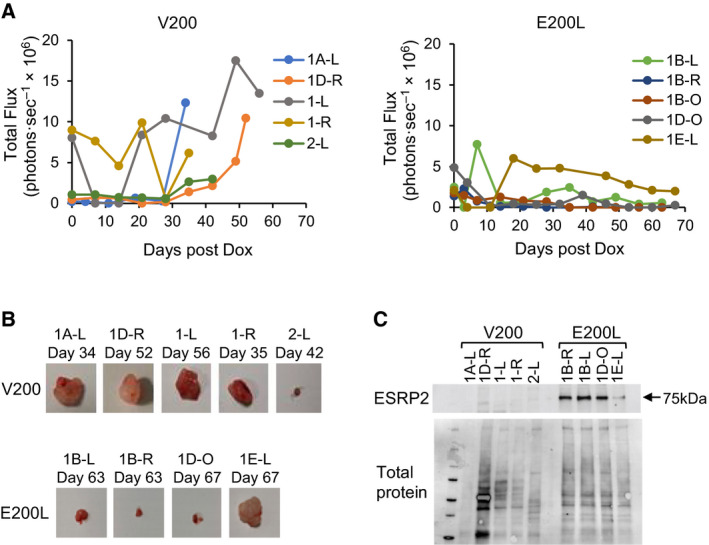
Tumorigenicity of *ESRP2*‐expressing WT cells. Orthotopic xenografts of V200 and E200L cells were produced by injecting cells under the kidney capsule of nude mice (*n* = 5 for each cell line). When a bioluminescent signal was detected above background (incipient growth of tumours), mice were injected intraperitoneally with doxycycline (Dox) three times per week, as described in Materials and Methods. (A) Time course of tumour growth as assayed by in vivo bioluminescence in V200 (left) and E200L (right) xenografts. Plots show tumour signals days after doxycycline induction (i.e. first doxycycline injection = day zero) for each individual mouse. Plot of the average of the bioluminescence traces is shown in Fig. S13A. (B) Tumours excised from mice (no tumour was excisable in mouse 1B‐0 at day 63, therefore only four E200L tumours are shown). Full details of tumour size and weight are shown in Fig. S13C. (C) Western blot of ESRP2 protein expression in excised tumours.

We used orthotopic xenografts of the Wit49‐derived cell lines, under the kidney capsule of nude mice [34] (Fig. 5), to examine the effect of ESRP2 expression *in vivo*. After treatment with doxycycline, tumours produced by V200 cells continued to proliferate, whilst tumours produced by E200L cells stopped growing, or regressed (Fig. 5A and Fig. S13A, B). V200 cells produced large tumours in four of five mice, but only one mouse out of five injected with E200L cells (1E‐L) produced a large tumour (Fig. 5B and Fig. S13C). Western blotting of excised tumours demonstrated that doxycycline treatment had induced high‐level ESRP2 expression in all E200L tumours, with the notable exception of 1E‐L (where the tumour grew larger) and V200‐induced tumours (Fig. 5C). This therefore demonstrated a strong correlation between ESRP2 expression and suppression of tumour growth.

### 3.6 RNA‐seq analysis of alternative splicing in Wilms tumour cell lines

In biological duplicates, we carried out RNA‐seq on E200L cells that were doxycycline‐induced (ESRP2‐expressing) or uninduced (non‐expressing), obtaining between 70 and 80 million paired‐end reads per sample. These reads were mapped onto the human genome, examined for differential gene expression and used in rMATS software [26] to identify alternative splicing events.

**Fig. 6:**
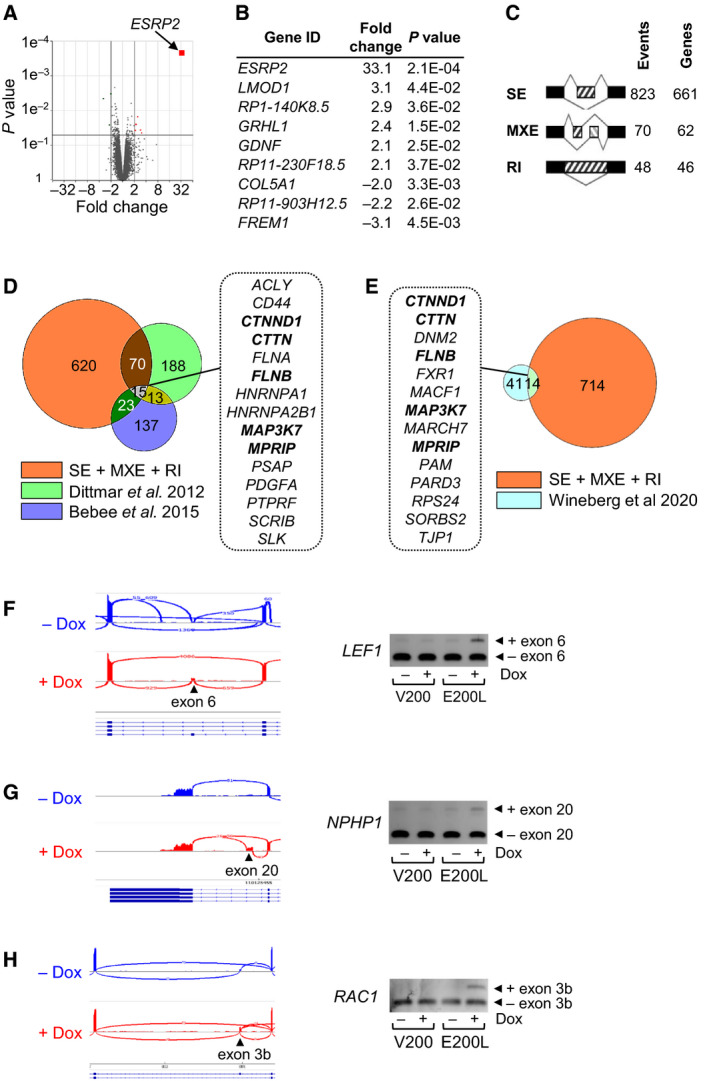
RNA‐seq analysis identifies ESRP2 targets in a WT cell line. RNA‐seq was performed on E200L cells with or without 96 h of doxycycline induction (to induce high‐level ESRP2 expression). (A) Volcano plot of *P* value versus fold induction of transcripts in ESRP2‐expressing E200L cells compared with non‐expressing cells. Genes induced > 2‐fold with *P* < 0.05 are indicated in red, and *ESRP2* is labelled. (B) List of genes in A that were induced > 2‐fold with *P* < 0.05. (C) Number of altered splicing events and affected genes induced by ESRP2 expression. SE; skipped exons, MXE; mutually exclusive exons; RI, retained introns. Tables S6 to S8 for full details. (D) Venn diagram comparing genes identified in this study (SE + MXE + RI) with two other RNA‐seq analyses of ESRP‐induced splicing changes [36, 37]. (E) Venn diagram comparing genes identified in this study (SE + MXE + RI) with an RNA‐seq analysis of MET‐associated splicing changes in the developing kidney [38]. (F, G and H) Alternative splicing of novel targets *LEF1* (F), *NPHP1* (G) and *RAC1* (H). Left‐hand panels: Sashimi plots of RNA‐seq data from E500L cells uninduced (‐Dox) or induced to express ESRP2 (+Dox). Right‐hand panels: Agarose gels of RT‐PCRs of amplicons spanning alternatively spliced exons (Table S1 for primers), in V200 and E200L cells, either uninduced, or doxycycline‐induced to produce high‐level ESRP2 expression in E200L cells.

Very few transcripts, apart from *ESRP2*, showed significant changes in RNA expression (*P* < 0.05, fold change > 2) when ESRP2 expression was induced (Fig. 6A, B). Interestingly, one induced gene was *GRHL1*, and grainyhead‐like transcription factors are important in both kidney development and MET [35], making them good candidates for an involvement in WT. However, we found no difference in expression of *GRHL1* between NT and WT (Fig. S14), which does not support a role for altered *GRHL1* expression in WT pathogenesis.

In contrast to the lack of altered gene transcription, ESRP2 induction was associated with over 900 splicing events involving over 700 genes, with significant changes (false discovery rate, FDR < 0.05) in skipped exons, mutually exclusive exons and retained introns (Fig. 6C, Table S6, S7, and S8). The genes involved were particularly enriched for biological processes concerned with vesicular and intracellular transport (Table S9). Although we found many ESRP target genes in common with other reports [36, 37], we also identified over 600 novel target genes (Fig. 6D). Comparison with a recent study of MET‐associated alternative splicing changes during kidney development [38] also revealed overlap with some of our target genes (Fig. 6E). Interestingly, the two lists of genes identified as overlapping our ESRP2 targets, included five genes (33–36%) in common (*CTNND1*, *CTTN*, *FLNB*, *MAP3K7* and *MPRIP*; shown in bold in Fig. 6D, E), emphasising the importance of ESRP‐regulated alternative splicing in kidney development.

We validated a selection of putative targets by specific RT‐PCR assays, to examine exon inclusion upon ESRP2 induction. We successfully validated several previously identified targets; *CD44*, *ENAH*, *FGFR2*, *SCRIB* and *SLK* (Fig. S15), as well as the novel targets *LEF1*, *NPHP1* and *RAC1* (Fig. 6F, G, H). However, some putative target genes showed no altered splicing after ESRP2 induction (Fig. S16 and Table S10).

**Fig. 7:**
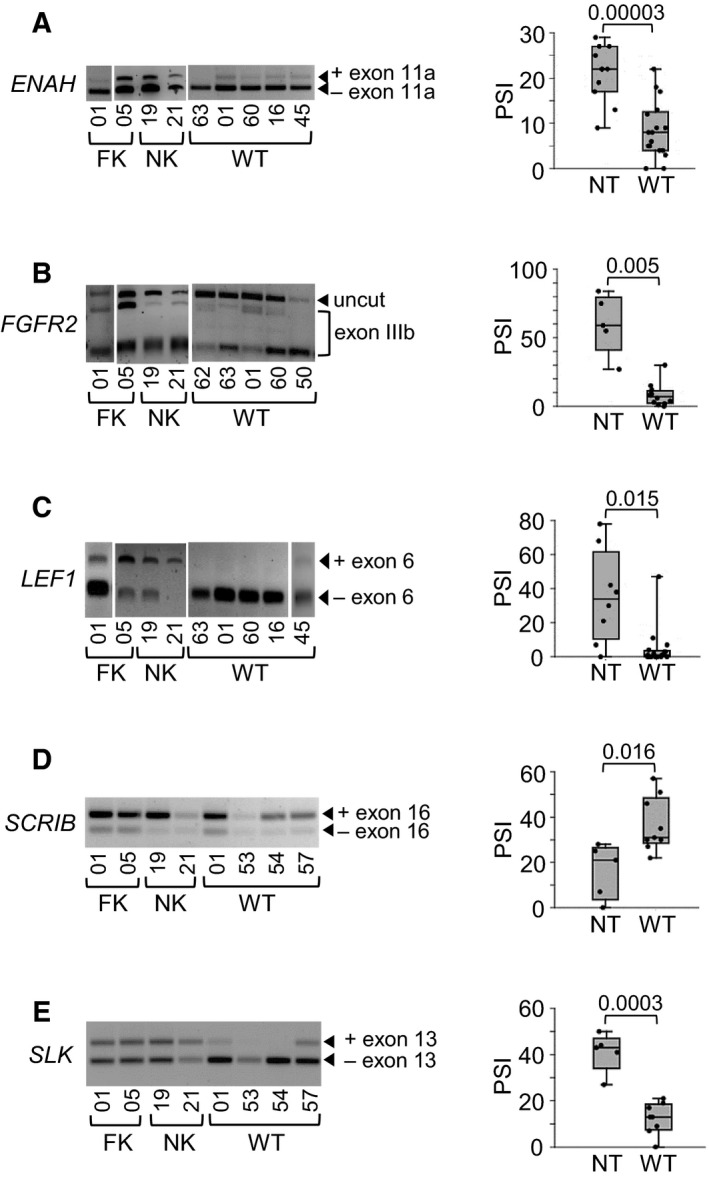
Alternative splicing of ESRP2 target genes in Wilms tumour. (A to E) Left‐hand panels: Representative agarose gels of RT‐PCRs of amplicons spanning the alternatively spliced exons (Table S1 for primers), from FK (foetal kidney), NK (normal kidney) and WT (Wilms tumour). *FGFR2* exon IIIb was detected by restriction digest with AvaI [24]. Right‐hand panels: Dot‐boxplots showing per cent splice inclusion (PSI) in NT (normal tissue) and WT. *P* values from *t*‐test. *ENAH* (A), NT *n* = 11 (4 FK and 7 NK), WT *n* = 17; *FGFR2* (B), NT *n* = 5 (3 FK and 2 NK), WT *n* = 12; *LEF1* (C), NT *n* = 8 (5 FK and 3 NK), WT *n* = 17; *SCRIB* (D), NT *n* = 5 (2 FK and 3 NK), WT *n* = 9; *SLK* (E), NT *n* = 5 (2 FK and 3 NK), WT *n* = 8.

To investigate the possible role of ESRP2 target genes in WT pathogenesis, we examined alternative splicing of 12 genes (seven novel and five previously described) in FK, NK and WT (Fig. 7 and Fig. S17). Five genes (42%) showed significant changes in the degree of alternative splicing between normal tissues and WT (Fig. 7), and seven (58%) did not (Fig. S17, Table S10).

## 4 Discussion

**Fig. 8:**
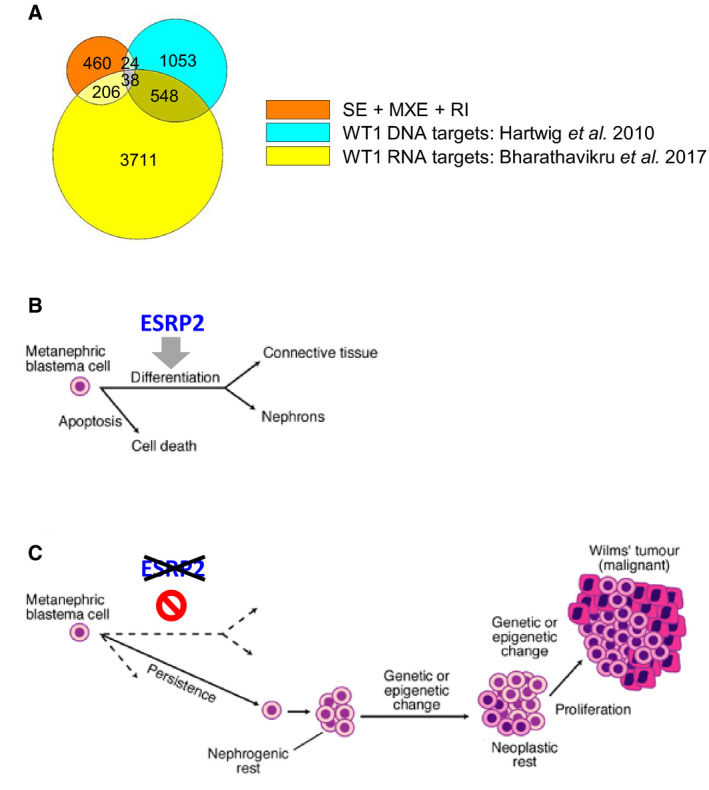
ESRP2 action in Wilms tumour. (A) Venn diagram comparing the 728 unique genes identified in this study (SE + MXE + RI, skipped exons; mutually exclusive exons and retained introns; Fig. 6) with 1663 WT1 DNA‐binding targets identified by chromatin immunoprecipitation in developing kidney [50] and 4503 WT1 RNA‐binding targets (protein‐coding genes) identified by RNA immunoprecipitation in M15 mesonephric cells [51]. (B) ESRP2 may be required for epithelial differentiation, to form nephrons during kidney development. (C) Loss of ESRP2 function by hypermethylation may inhibit normal differentiation and therefore promote persistence of undifferentiated blastema, leading to nephrogenic rest formation and eventual progression to Wilms tumour. B and C adapted from Fig. 2 in reference [1].

This is the first demonstration of *ESRP2* repression caused by DNA hypermethylation in WT, which implicates RNA splicing alterations as an important pathogenic factor in WT development. Investigation of matched sets of NK, NR and WT (Fig. 2D, E) suggested that inactivation of *ESRP2* by DNA methylation occurs at an early stage in kidney development, prior to NR formation. We propose that ESRP2 is essential for the differentiation of the metanephric blastema into nephrons (Fig. 8B) and that loss of ESRP2 expression causes a differentiation block, initiating NRs, that can undergo further genetic and epigenetic defects to produce WT (Fig. 8C). Support for this model comes from studies showing that the Esrp paralogs are expressed in the developing kidney [39], with increased expression of Esrp 1 and 2 when renal precursors undergo epithelial differentiation [38], and that knockout of Esrp genes in mice decreases kidney volume, due to a lack of nephrons [40].

Inactivation of *ESRP2* as an early premalignant event in WT development probably explains why we found no association with clinical features (Figs S5 and S6). It also explains why we found no association between *ESRP2* methylation and LOH at 16q (Fig. S5C), where the ESRP2 gene is located, because we have previously demonstrated that 16q LOH occurs after NR formation [14], that is after *ESPR2* hypermethylation.

We have shown that *ESRP1*, though an *ESRP2* paralogue, is not repressed by hypermethylation in WTs (Fig. 2B, C and Fig. 3C, D). This implies that *ESRP1* and *ESRP2* may have different biological functions and are regulated differently in some instances, as recently reported in prostate cancer, where *ESRP2* but not *ESRP1* is regulated by androgens [41].

Splicing alterations are frequent in human cancers [42], including ESRP‐induced changes in breast cancer [33, 43], prostate cancer [41, 44], renal cell carcinoma [45] and colorectal cancer [46]. Most studies have reported expression changes without finding underlying genetic or epigenetic defects in the *ESRP* genes themselves [33, 41, 43, 45, 46]. However, there are reports of genetic defects in *ESRP* genes in human cancers, specifically, microsatellite indels [47] or duplications [44] of *ESRP1*. In addition, there are reports of DNA methylation changes in *ESRP1* in prostate cancer [48] and of *ESRP2* in breast cancer [49], and our examination of TCGA data (Fig. S9D) demonstrated *ESRP2* methylation changes in several other adult cancer types. Thus, our results add to a growing body of evidence that *ESRP* genes can be either genetically or epigenetically deregulated in a wide range of human cancers.

Our functional studies suggested that the main biological effect of ESRP2 is to regulate cell proliferation by slowing cell division (Fig. 4E–H and Fig. S11). Whilst we observed some actin cytoskeleton rearrangement (Fig. 4D), we did not observe significant expression changes in classical epithelial marker genes (Fig. 6A, B), nor any changes in cell motility or invasion (Fig. S12), unlike what occurs when ESRP expression is modulated in adult human cancer cell lines [30, 32, 33]. Coupled with our xenograft experiments that identify *ESRP2* as a *bona fide* tumour suppressor gene (Fig. 5), these results suggest that the tumour suppressor activity of *ESRP2* in WT cell lines occurs mainly by altering cell growth properties, rather than by affecting cellular differentiation.

Mechanistically, our RNA‐seq results demonstrated that ESRP2 modulated the splicing of a diverse range of genes, including both well‐established and novel targets (Fig. 6 and Tables S6 to S8). A subset of these genes showed reduced expression of their epithelial splice forms in WT (Fig. 7), consistent with DNA hypermethylation‐induced downregulation of *ESRP2* in WT (Fig 2 and Fig. 3). Interestingly, of the 728 genes that we identified as having their splicing modulated by ESRP2 (Fig. 6), only 62 (9%) are WT1 DNA‐binding targets [50], whereas 244 (34%) are WT1 RNA‐binding targets [51] (Fig. 8A). The WT1 RNA‐binding targets include all five of the ESRP2‐regulated genes that we found in common between our results and two other RNA‐seq studies (Fig. 6D, E). This suggests that WT1 and ESRP2 are involved in the post‐transcriptional regulation of a similar set of genes during renal development. Since *ESRP2* hypermethylation is an early event, like *WT1* mutation [52, 53], this suggests that *ESRP2* hypermethylation may be another important early event in WT development, which contributes to WT pathogenesis by inhibiting MET (Fig. 8C). These results, together with genetic evidence showing defects in miRNA‐processing genes in WT [8, 9, 10, 11, 12], reinforce the critical role that post‐transcriptional gene regulation plays in WT pathogenesis.

## 5 Conclusions

Our genome‐wide DNA methylation analysis of WT has identified *ESRP2* as a novel differentially methylated gene. *ESRP2* was frequently silenced by DNA hypermethylation in WT, and this occurred early in WT development (in nephrogenic rests). *ESRP2* inhibited cellular proliferation *in vitro*, and *in vivo* it suppressed tumour growth of orthotopic xenografts in nude mice, demonstrating that *ESRP2* acts as a tumour suppressor gene in WT. Using RNA‐seq of the *ESRP2*‐expressing WT cell lines, we have identified several novel splicing targets, some of which affect pathways known to be important in kidney development. We propose that epigenetic inactivation of *ESRP2* disrupts the regulation of alternative splicing during the mesenchymal to epithelial transition in early kidney development, to generate WT.

## Conflict of interest

The authors declare no conflict of interest.

## Author contributions

DL carried out the RNA‐seq, target validation and other experimental work; LL performed the animal experiments; WM carried out pyrosequencing, qPCR and derived the inducible cell line; D Lee performed the bioinformatic analysis of the RNA‐seq data; MS performed the MCIP DNA methylation analysis; AZ, LP and WYC performed DNA methylation and expression analyses on cell lines and tumours; YA and JB validated putative ESRP2 targets; RW and KPJ provided tumour samples and clinical data; KTAM help devise the methylation analysis strategy; SO planned experiments, especially the animal work; and KWB wrote the paper, planned work and carried out some experimental work. All authors viewed the manuscript and were given the opportunity to comment on it.

### Peer Review

The peer review history for this article is available at https://publons.com/publon/10.1002/1878‐0261.13101.

## Supporting information


**Fig. S1**. Cell line STR profiles.
**Fig. S2**. ESRP2 inducible expression construct.
**Fig. S3**. ESRP2 methylation detected by MCIP.
**Fig. S4**. ESRP2 methylation and RNA expression in Wilms tumours from two cohorts.
**Fig. S5**. ESRP2 DNA methylation in Wilms tumours of different stages and 16q LOH status.
**Fig. S6**. Overall survival in Wilms tumour patients with different levels of ESRP2 methylation.
**Fig. S7**. ESRP2 methylation and expression in Wilms tumours of different histological subtypes.
**Fig. S8**. Hierarchical clustering of Wilms tumours by DNA methylation.
**Fig. S9**. ESRP2 methylation in other childhood renal tumours and adult cancers.
**Fig. S10**. Transient transfection of Wilms tumour cells with Esrp1 and Esrp2.
**Fig. S11**. Growth of Wit49 transfected cells.
**Fig. S12**. Motility assays of Wit49 transfected cells.
**Fig. S13**. Mouse tumorigenicity data.
**Fig. S14**. GRHL1 RNA expression.
**Fig. S15**. Successfully validated putative ESRP2 target genes.
**Fig. S16**. Unsuccessfully validated putative ESRP2 target genes.
**Fig. S17**. Alternative splicing of putative ESRP2 target genes in normal kidney and Wilms tumour.

**Table S1**. Oligonucleotide primers used for PCR.
**Table S2**. MCIP hits and filtering.
**Table S3**. Pyrosequencing assays.
**Table S4**. MCIP hits GO analysis.
**Table S5**. Properties of tumours used in Fig. S8.
**Table S6**. Skipped exons (SE) identified with rMATs.
**Table S7**. Mutually exclusive exons (MXE) identified with rMATS.
**Table S8**. Retained introns (RI) identified with rMATS.
**Table S9**. GO analysis of all genes identified with rMATS from RNA‐seq data; Mutually exclusive exons (MXE), Retained introns (RI) and Skipped exons (SE).
**Table S10**. Putative ESRP2 targets.

## Data Availability

Supporting data are available in the supplementary material of this article [Figs S1 to S17, and Tables S1 to S10]. The genome‐wide DNA methylation (MCIP) data that support the findings of this study are openly available in NCBI's Gene Expression Omnibus and are accessible through https://www.ncbi.nlm.nih.gov/geo/query/acc.cgi?acc=GSE153047, GEO Series accession number GSE153047. The RNA‐seq data that support the findings of this study are openly available in NCBI's Gene Expression Omnibus and are accessible through https://www.ncbi.nlm.nih.gov/geo/query/acc.cgi?acc=GSE154496, GEO Series accession number GSE154496.
