# Supplementary material for: The epithelial splicing regulator ESRP2 is epigenetically repressed by DNA hypermethylation in Wilms tumour and acts as a tumour suppressor
Source: Mol Oncol. 2021 Sep 28;16(3):630–47. doi: 10.1002/1878-0261.13101 (PMC8807366; doi:10.1002/1878-0261.13101)
Supplement: Supplementary file 1 — Fig. S1. Cell line STR profiles. Fig. S2. ESRP2 inducible expression construct. Fig. S3. ESRP2 methylation detected by MCIP. Fig. S4. ESRP2 methylation and RNA expression in Wilms tumours from two cohorts. Fig. S5. ESRP2 DNA methylation in Wilms tumours of different stages and 16q LOH status. Fig. S6. Overall survival in Wilms tumour patients with different levels of ESRP2 methylation. Fig. S7. ESRP2 methylation and expression in Wilms tumours of different histological subtypes. Fig. S8. Hierarchical clustering of Wilms tumours by DNA methylation. Fig. S9. ESRP2 methylation in other childhood renal tumours and adult cancers. Fig. S10. Transient transfection of Wilms tumour cells with Esrp1 and Esrp2. Fig. S11. Growth of Wit49 transfected cells. Fig. S12. Motility assays of Wit49 transfected cells. Fig. S13. Mouse tumorigenicity data. Fig. S14. GRHL1 RNA expression. Fig. S15. Successfully validated putative ESRP2 target genes. Fig. S16. Unsuccessfully validated putative ESRP2 target genes. Fig. S17. Alternative splicing of putative ESRP2 target genes in normal kidney and Wilms tumour. [file MOL2-16-630-s001.pdf]

Legge et al

The epithelial splicing regulator *ESRP2* is epigenetically repressed by DNA hypermethylation in Wilms tumour and acts as a tumour suppressor

Supplementary data: Figures S1 to S17

**S1**	Cell line STR profiles
**S2**	*ESRP2* inducible expression construct
**S3**	*ESRP2* methylation detected by MCIP
**S4**	*ESRP2* methylation and RNA expression in Wilms tumours from two cohorts
**S5**	*ESRP2* DNA methylation in Wilms tumours of different stages and 16q LOH status
**S6**	Overall survival in Wilms tumour patients with different levels of *ESRP2* methylation
**S7**	*ESRP2* methylation and expression in Wilms tumours of different histological subtypes
**S8**	Hierarchical clustering of Wilms tumours by DNA methylation
**S9**	*ESRP2* methylation in other childhood renal tumours and adult cancers
**S10**	Transient transfection of Wilms tumour cells with Esrp1 and Esrp2
**S11**	Growth of Wit49 transfected cells
**S12**	Motility assays of Wit49 transfected cells
**S13**	Mouse tumorigenicity data
**S14**	*GRHL1* RNA expression
**S15**	Successfully validated putative ESRP2 target genes
**S16**	Unsuccessfully validated putative ESRP2 target genes
**S17**	Alternative splicing of putative ESRP2 target genes in normal kidney and Wilms tumour

A

Locus	Wit49 Yeger	Wit49	17.94
AM	X	X, X	X
D3S1358	17	17, 17	16
D1S1656		12, 16	
D6S1043		11, 12	
D13S317	11, 12	11, 12	8
Penta E	13	13, 13	7, 11
D16S539	12	12, 12	11
D18S51	15	15, 15	14, 17
D2S1338		23, 26	
CSF1PO	11, 12	11, 12	11, 12
Penta D	11, 12	11, 12	9, 11
TH01	9.3	9.3, 9.3	8, 9.3
vWA	16, 18	16, 18	14, 16
D21S11	29, 32.2	29, 32.2	29
D7S820	8, 11	8, 11	9, 11
D5S818	12, 13	12, 13	11
TPOX	8	8, 8	8
D8S1179	10	10, 10	10, 13
D12S391		20, 23	
D19S433		14, 14	
FGA	25	25, 25	22, 23

B

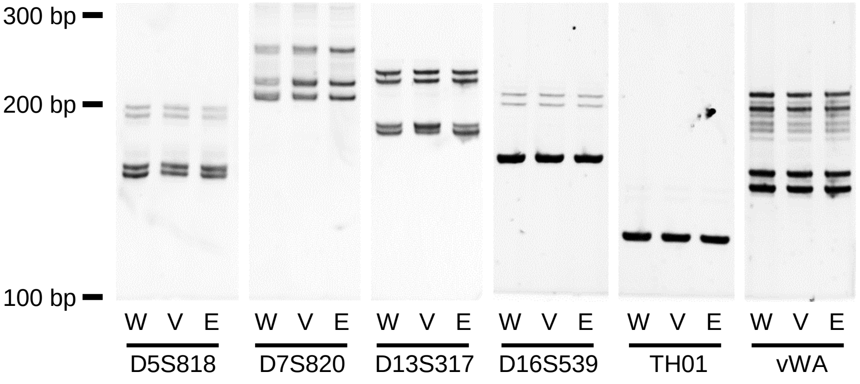

Figure S1: Cell line STR profiles

A: STR profiles for the original Wit49 cell line (personal communication Prof. H Yeger), Wit49 (carried out by Eurofins; https://www.eurofinsgenomics.eu/) and 17.94 (carried out by DSMZ; https://www.dsmz.de/) used in our laboratory (Bristol) for this paper.

B: Six STR markers amplified by PCR (supplementary table S1) and run on 7% non-denaturing acrylamide gels. Original Wit49 cells (W), and the V200 (V) and E200L (E) derivatives used in this paper all show identical profiles, with the STR amplicons having the predicted sizes from the previous STR profiling listed in A.

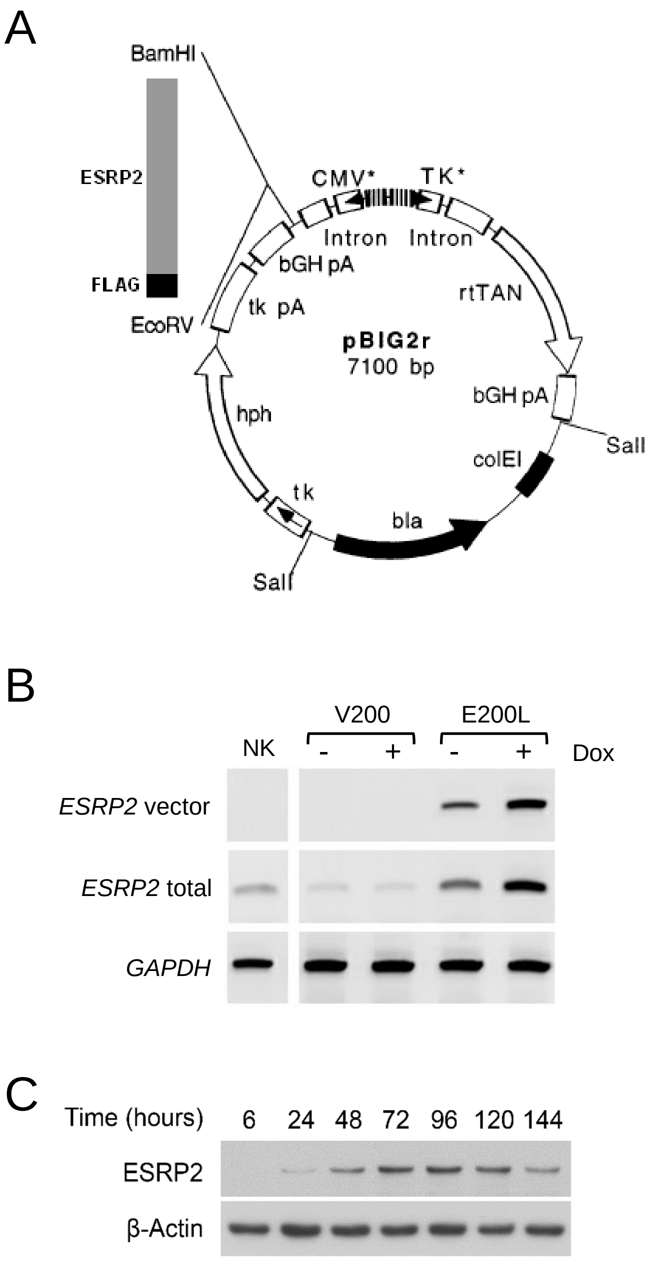

Figure S2: *ESRP2* inducible expression construct

A: *ESRP2* cDNA was amplified by PCR from IMAGE clone 4810948, using a forward primer containing a BamHI site and a reverse primer containing an EcoRV site plus a FLAG tag (supplementary table S1). This insert was then ligated into BamHI/EcoRV-digested pBIG2r (Strathdee, C. A. et al (1999) Gene 229(1-2): 21-29).

B: RT-PCR of cDNA from normal kidney (NK), control vector-transfected Wit49 cells (V200) and *ESRP2*-transfected Wit49 cells (E200L), amplified with primers for *ESRP2* expressed from the vector (ESRP2 RTF2 and BGHR), for total *ESRP2* (ESRP2 RTF2 and ESRP2 RTR2) and for *GAPDH* (GAPDH F and GAPDH R). Primer BGHR is located in the bGH pA region in the pBIG2R vector downstream of the insert (see A above) and the *ESRP2* primers are in the cDNA (supplementary table S1).

C: Western blot showing time course of ESRP2 protein expression induced in cell line E200L with 2 µg/ml doxycycline.

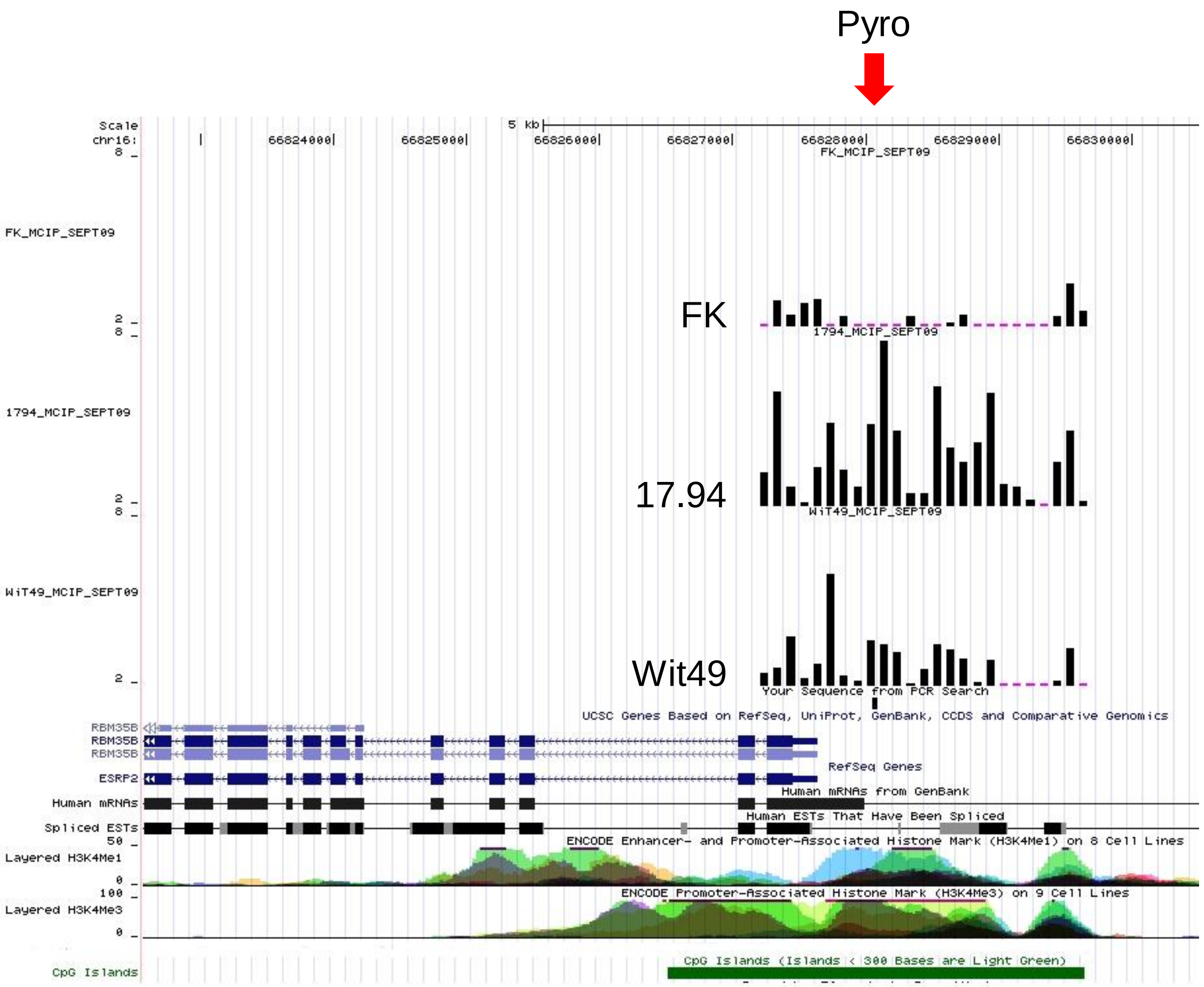

Figure S3: *ESRP2* methylation detected by MCIP
Black bars show the probe ratios derived from MCIP for fetal kidney (FK) and the two WT cell lines, 17.94 and Wit49, positioned on the *ESRP2* gene, showing the transcripts (*ESRP2* / *RBM35B*), H3K4Me1 and Me3 marks (ENCODE) and CpG islands (http://genome.ucsc.edu). The position of the pyrosequencing assay is indicated by the red arrow shown at the top.

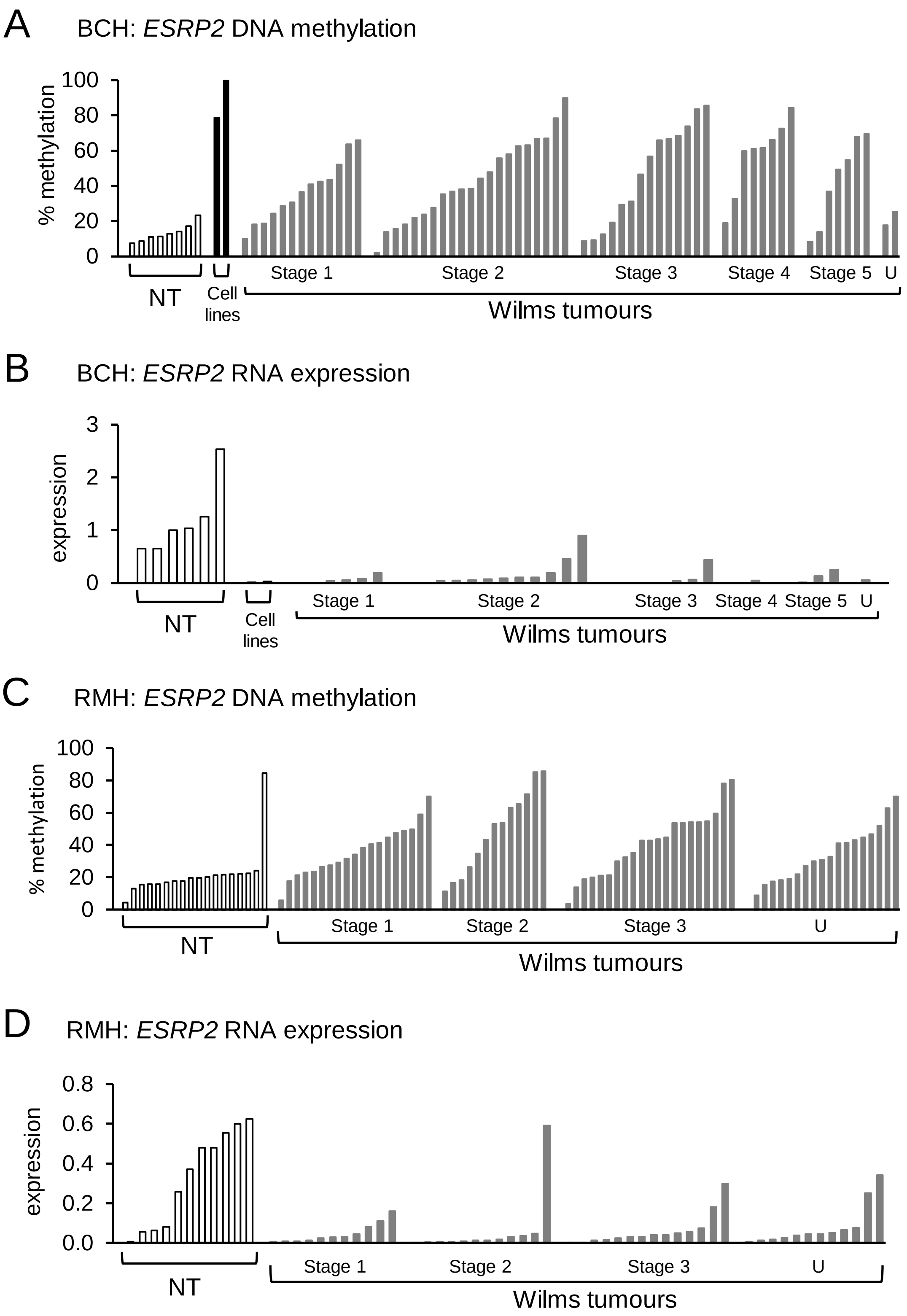

Figure S4: *ESRP2* methylation and RNA expression in Wilms tumours from two cohorts

A: *ESRP2* DNA methylation versus stage (BCH data; pyrosequencing).
B: *ESRP2* RNA expression versus stage (BCH data; QPCR).
C: *ESRP2* DNA methylation versus stage (RMH data; pyrosequencing).
D: *ESRP2* RNA expression versus stage (RMH data; QPCR).
NT, normal tissues; U, stage unknown.

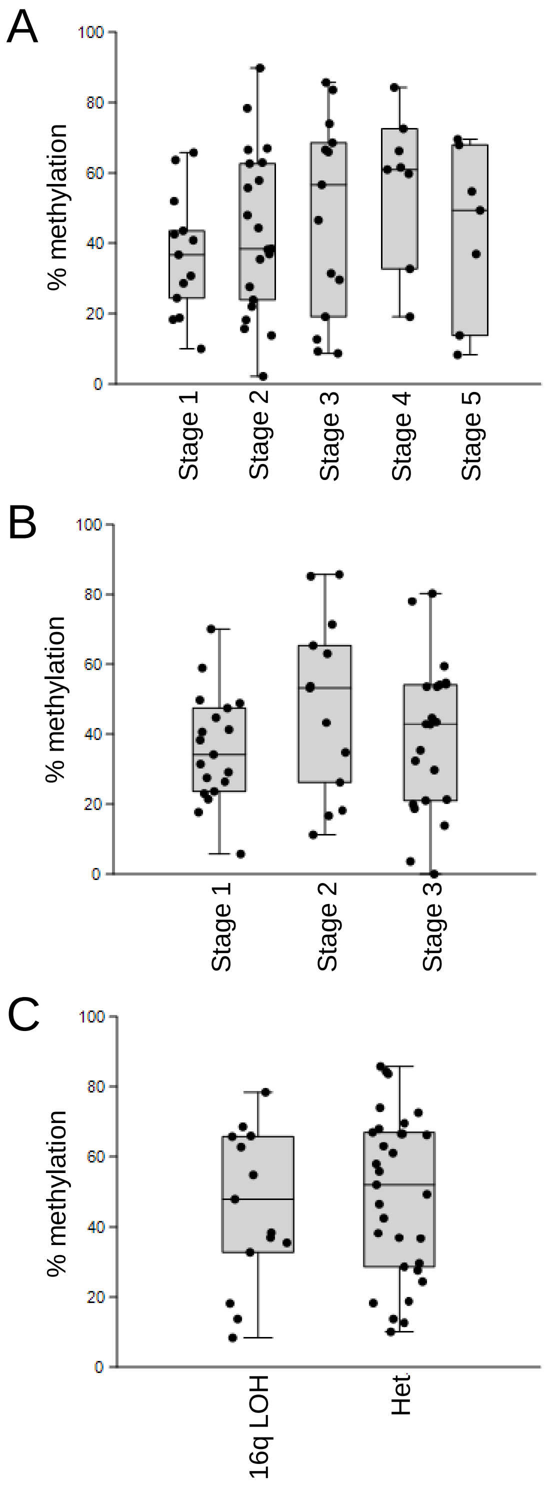

Figure S5: *ESRP2* DNA methylation in Wilms tumours of different stages and 16q LOH status

A: Dot-Boxplot of *ESRP2* DNA methylation versus stage (BCH data; for stages 1, 2, 3, 4, 5; n = 16, 24, 16, 9, 8 respectively).

B: Dot-Boxplot of *ESRP2* DNA methylation versus stage (RMH data; for stages 1, 2, 3; n = 13, 7, 16 respectively).

C: Dot-Boxplot of *ESRP2* DNA methylation versus 16q loss of heterozygosity (BCH data; LOH, n = 8; Het, n = 22). LOH, loss of heterozygosity; Het, heterozygous tumours.

ESRP2 DNA methylation was assayed by pyrosequencing.

A

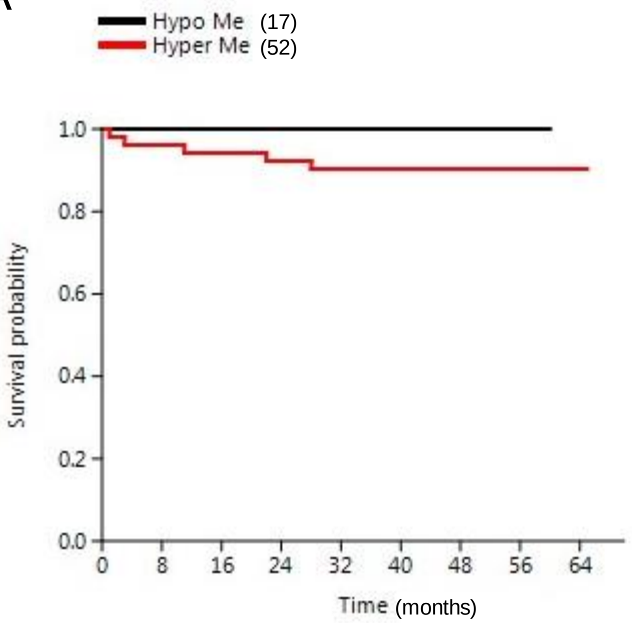

BCH

B

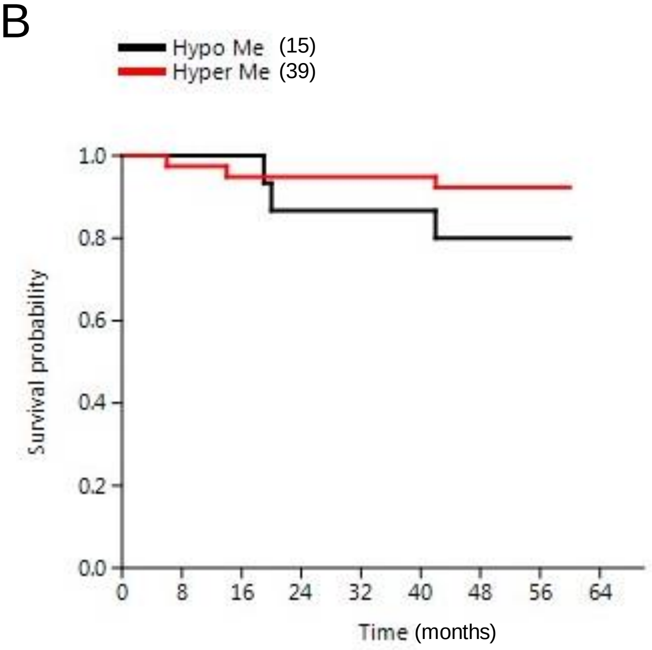

RMH

Figure S6: Overall survival in Wilms tumour patients with different levels of *ESRP2* methylation

A: Overall patient survival up to 5 years post diagnosis – BCH cohort. p = 0.200
B: Overall patient survival up to 5 years post diagnosis – RMH cohort. p = 0.212
Hypo Me, hypomethylated tumours (*ESRP2* DNA methylation <25%).
Hyper Me, hypermethylated tumours (*ESRP2* DNA methylation >25%).
ESRP2 DNA methylation was assayed by pyrosequencing.
p values calculated using log-rank test.

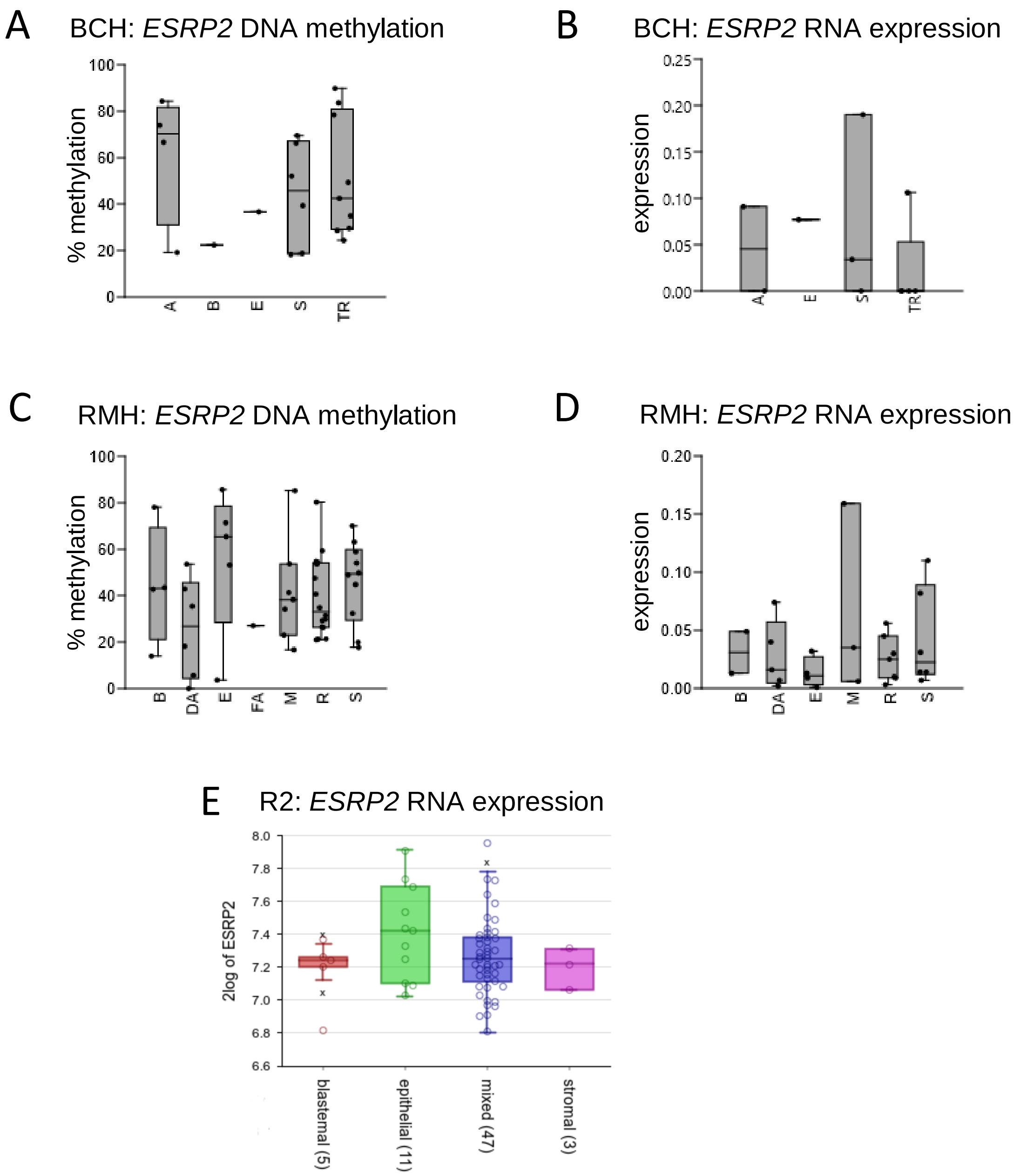

Figure S7: *ESRP2* methylation and expression in Wilms tumours of different histological subtypes

A, B: Dot-Boxplots of *ESRP2* DNA methylation (**A**) and RNA expression (**B**) in Wilms tumours from the BCH cohort, classified by histology. A = anaplastic, B = blastemal predominant, E = epithelial predominant, S = stromal predominant, TR = triphasic. **C, D:** Dot-Boxplots of *ESRP2* DNA methylation (**C**) and RNA expression (**D**) in Wilms tumours from the RMH cohort, classified by histology. B = blastemal predominant, DA = diffuse anaplastic, E = epithelial predominant, FA = focal anaplastic, M = mixed histology, R = regressive, S = stromal predominant. In A to D, DNA methylation was assayed by pyrosequencing and RNA expression by QPCR. There were no significant differences between the different histological subtypes in A to D (Tukey's pairwise test).

E: *ESRP2* RNA expression from Affymetrix U133a arrays, in dataset "Tumor Wilms FHWT – Perlman" from R2 (https://r2.amc.nl). There were no significant differences between the different histological subtypes (ANOVA).

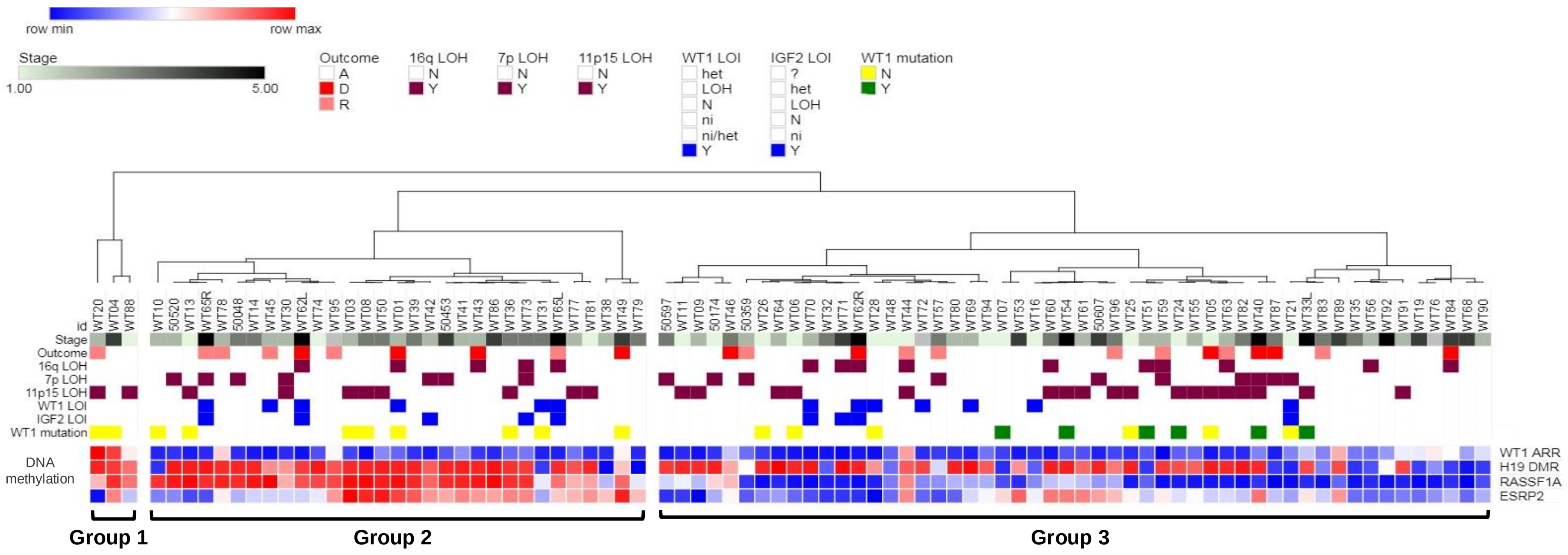

Figure S8: Hierarchical clustering of Wilms tumours by DNA methylation

WTs from the BCH cohort were assayed for DNA methylation by pyrosequencing at the *WT1* antisense regulatory region (ARR), the *H19* differentially methylated region (DMR), the *RASSF1A* promoter and the *ESRP2* promoter. Details of the pyrosequencing assays are given in supplementary table S3 and full methylation results in supplementary table S5. Loss of heterozygosity (LOH), loss of imprinting (LOI), stage and survival data taken from Powlesland, R. M. et al (2000) British Journal of Cancer 82:323-329 and Brown, K. W. et al (2008) Mol Cancer Res 6:1114-1123. *WT1* mutation data from Brown, K. W. et al (1992) Oncogene 7:763-768, Brown, K. W. et al (1993) Gene Chromosome Cancer 8:74-79, Miyagawa, K. et al (1998) Nat Genet 18:15-17 and Charles, A. K. et al (1998) Am J Pathol 153:991-1000.

Samples were clustered by one minus Pearson correlation using Morpheus software: https://software.broadinstitute.org/morpheus/

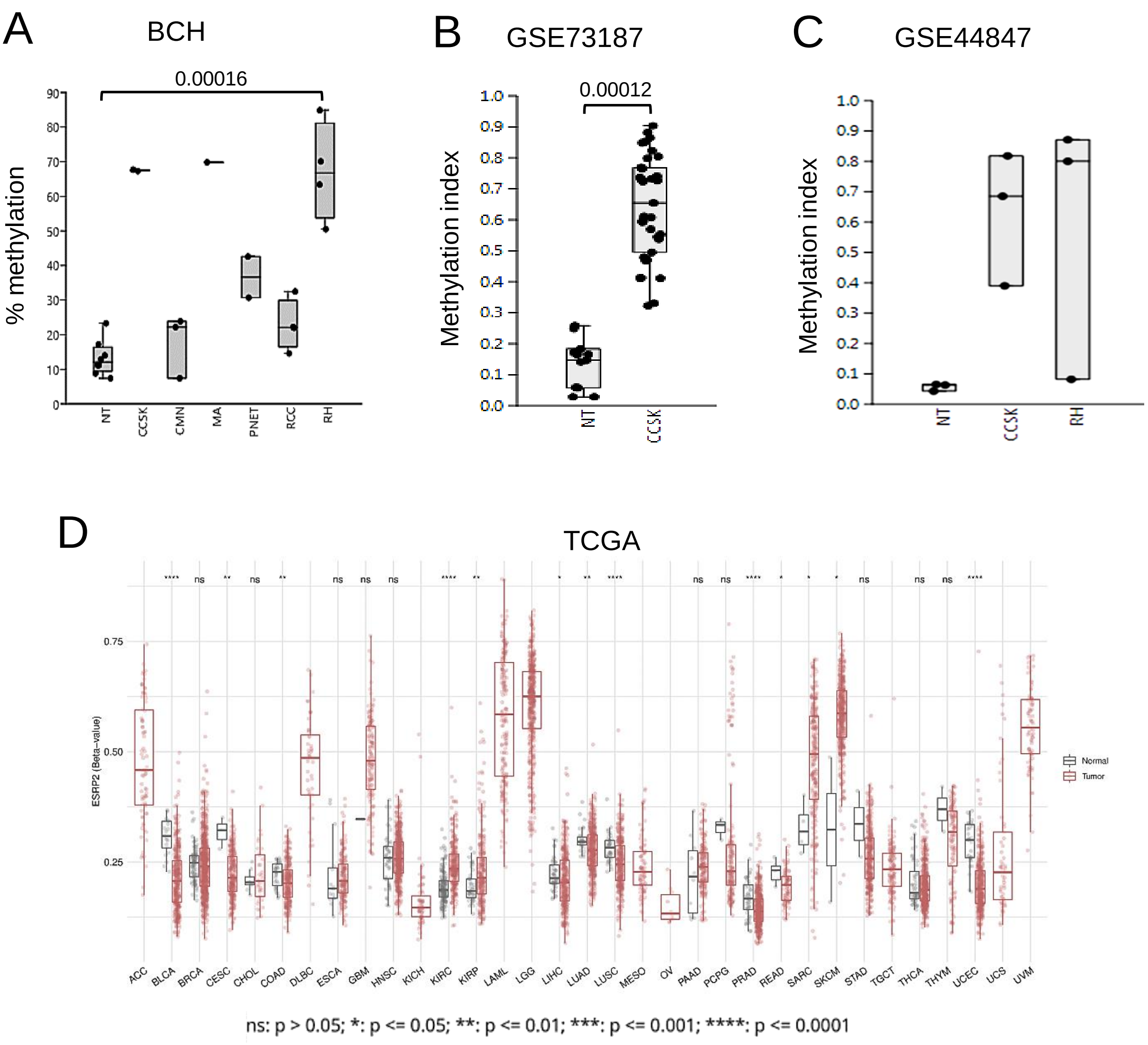

Figure S9: *ESRP2* methylation in other childhood renal tumours and adult cancers

A: Dot-Boxplot of DNA methylation of *ESRP2* assayed by pyrosequencing in eight normal tissue (NT) samples (four fetal kidneys and four normal kidneys), two clear cell sarcomas (CCSK), three mesoblastic nephromas (CMN), one metanephric adenoma (MA), two primitive neuroectodermal tumours (PNET), four renal cell carcinomas (RCC) and four rhabdoid tumours (RH), from local samples (BCH).

B: Dot-Boxplot of methylation data from GSE73187 of 12 normal tissue (NT) samples (four fetal kidneys and eight normal kidneys) and 34 CCSKs.

C: Dot-Boxplot of methylation data from GSE44847 of three normal kidney (NT) samples, three CCSKs and three RHs.

In B and C samples were assayed by Illumina Human Methylation 27 BeadChip arrays. Data shown is from cg20264732, which is in the 5' CpG island of *ESRP2*.

p value in A from Tukey's pairwise test, p value in B from t test.

D: *ESRP2* methylation in the TCGA dataset obtained using Shiny Methylation Analysis Resource Tool (SMART), http://www.bioinfo-zs.com/smartapp/.

Cancer abbreviations: ACC, Adrenocortical carcinoma; BLCA, Bladder Urothelial Carcinoma; BRCA, Breast invasive carcinoma; CESC, Cervical squamous cell carcinoma and endocervical adenocarcinoma; CHOL, Cholangiocarcinoma; COAD, Colon adenocarcinoma; DLBC, Diffuse Large B-cell Lymphoma; ESCA, Esophageal carcinoma; GBM, Glioblastoma multiforme; HNSC, Head and Neck squamous cell carcinoma; KICH, Kidney Chromophobe; KIRC, Kidney renal clear cell carcinoma; KIRP, Kidney renal papillary cell carcinoma; LAML, Acute Myeloid Leukemia; LGG, Brain Lower Grade Glioma; LIHC, Liver hepatocellular carcinoma; LUAD, Lung adenocarcinoma; LUSC, Lung squamous cell carcinoma; MESO, Mesothelioma; OV, Ovarian serous cystadenocarcinoma; PAAD, Pancreatic adenocarcinoma; PCPG, Pheochromocytoma and Paraganglioma; PRAD, Prostate adenocarcinoma; READ, Rectum adenocarcinoma; SARC, Sarcoma; SKCM, Skin Cutaneous Melanoma; STAD, Stomach adenocarcinoma; TGCT, Testicular Germ Cell Tumors; THCA, Thyroid carcinoma; THYM, Thymoma; UCEC, Uterine Corpus Endometrial Carcinoma; UCS, Uterine Carcinosarcoma; UVM, Uveal Melanoma.

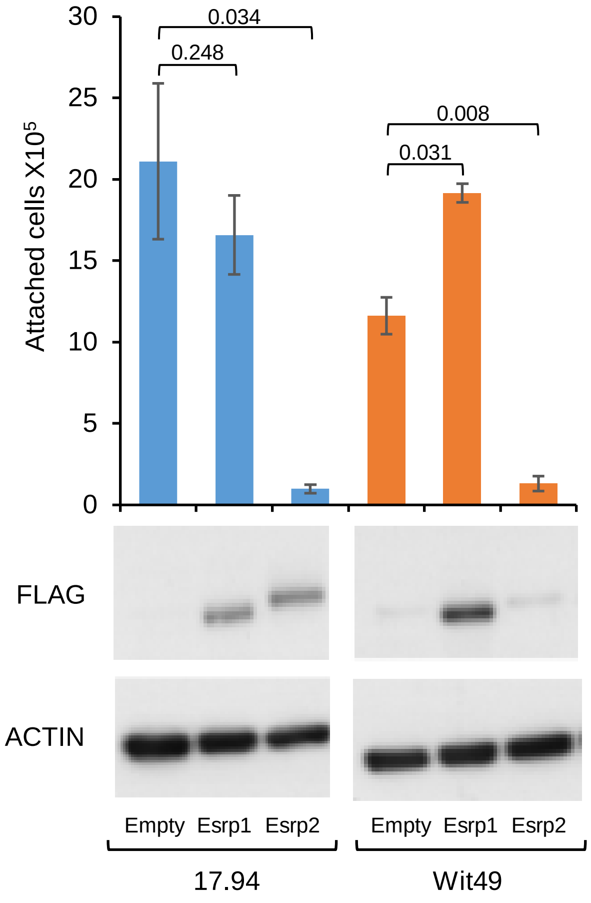

Figure S10: Transient transfection of Wilms tumour cells with Esrp1 and Esrp2
Wilms tumour cell lines 17.94 and Wit49 were transfected with plasmids expressing *Esrp1* or *Esrp2* cDNA (FLAG-tagged) or with an empty vector (Empty), and cell numbers counted after five days, as described in Material and Methods section 2.3. Chart show cell numbers (means ± SD of n=3) with Western blots below detecting FLAG-tagged Esrp1 and Esrp2, with Actin as a loading control. p values from t-test (Bonferroni-corrected).

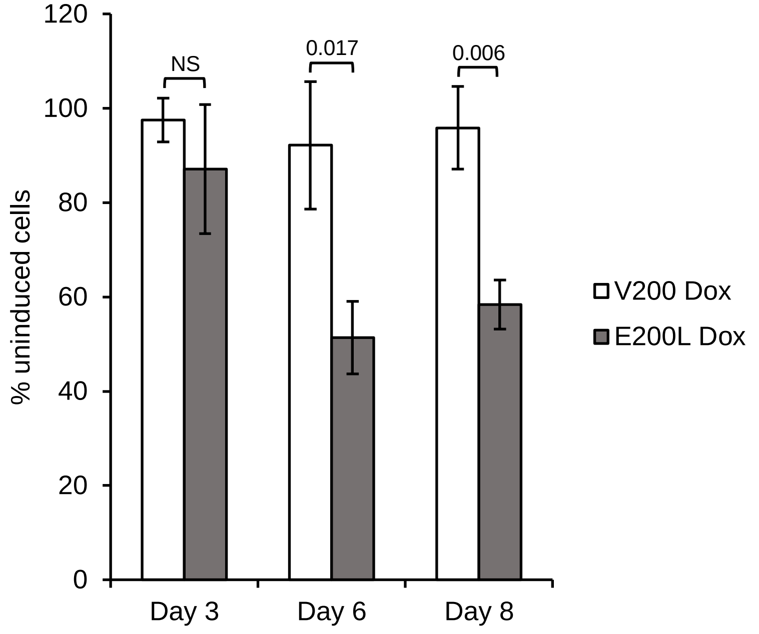

Figure S11: Growth of Wit49 transfected cells

Empty vector-transformed cells (V200) and *ESRP2*-transfected cells (E200L) were plated with and without doxycycline (2 µg/ml to induce *ESRP2* expression) and counted after 3, 6 and 8 days. Counts shown are the means ± SD of three experiments, expressed as the percentage of the uninduced cells at each time point. Cell growth was significantly reduced at 6 days and at 8 days (t-test) in E200L cells compared to V200 control cells.

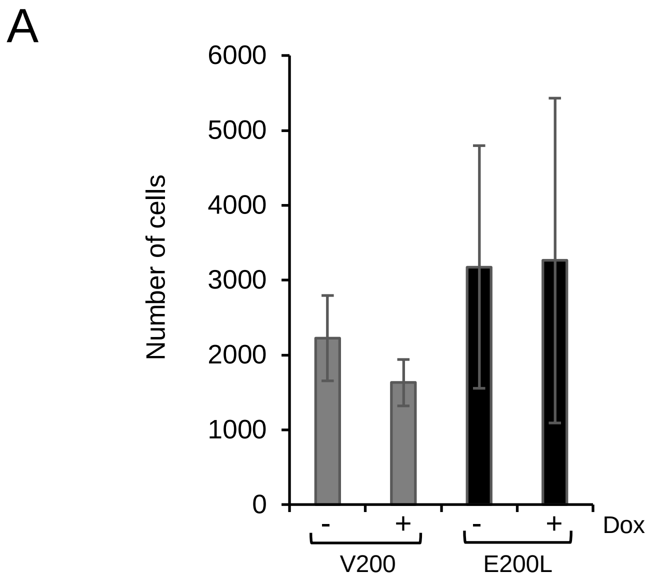

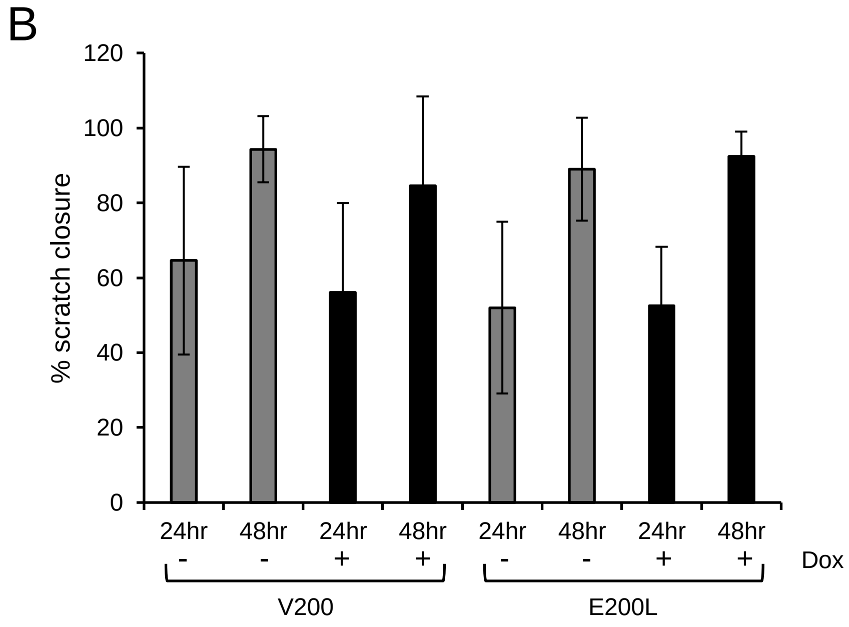

Figure S12: Motility assays of Wit49 transfected cells
A: Transwell assay of cell invasion; Cells were pre-treated for 4 days with 2 µg/ml Dox or control media, then seeded into transwell inserts in FBS-free DMEM and the inserts were put into wells filled with 10% FBS DMEM to produce a chemotactic gradient. Following 24 hours, inserts were washed, cells were fixed, stained and counted manually using light microscopy. Counts shown are the means ± SD of three experiments.
B: Scratch assay of cell motility; cells were seeded into 24 well plates and treated for 5 days with 2 µg/ml Dox or control media prior to a scratch being performed manually in the centre of each well. Wells were washed gently with PBS to remove dead cells from the scratch wound site. Control/Dox media was replaced and wells were analysed at 24 and 48 hours via widefield microscopy and Image J software to determine percentage wound closure. Counts shown are the means ± SD of three experiments.

A

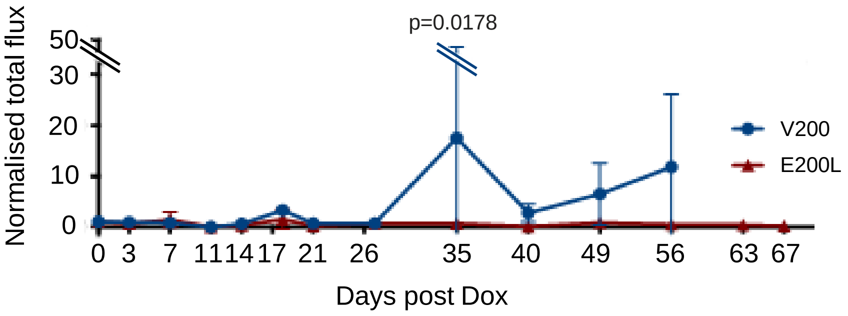

B

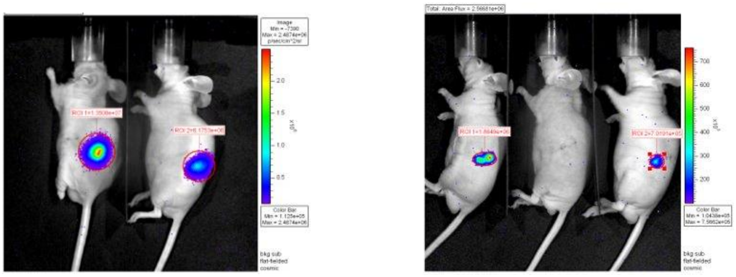

V200: Day 56 and 35

E200L: Day 63

C

Group	Mouse ID	Days after Doxy induction	Tumour width (mm)	Tumour length (mm)	Tumour volume (mm^3)	Tumour Weight (mg)
V200 (Control)	1A-L	34	10	11	1155	345
	1D-R	52	9	10	855	274
	1-L	56	8	10	720	155
	1-R	35	5	8	260	127
	2-L	42	2	2	8	7
E200L (ESRP2)	1B-L	63	3	4	42	35
	1B-R	63	2	3	15	8
	1D-O (two tumours)	67	1	2	3	5
			2	3	15	9
	1E-L	67	9	10	855	257

Figure S13: Mouse tumorigenicity data
A: Time course of tumour growth as assayed by in vivo bioluminescence. Plot shows the average ± SEM of tumour signals normalized to initial signal (i.e. the start of Dox induction); p=0.0178 by two-way ANOVA. Full traces of individual tumour growth are shown in figure 5A. **B:** Examples of *in vivo* bioluminescence imaging of nude mice carrying V200 and E200L xenografts. **C:** Size and weight of excised tumours.

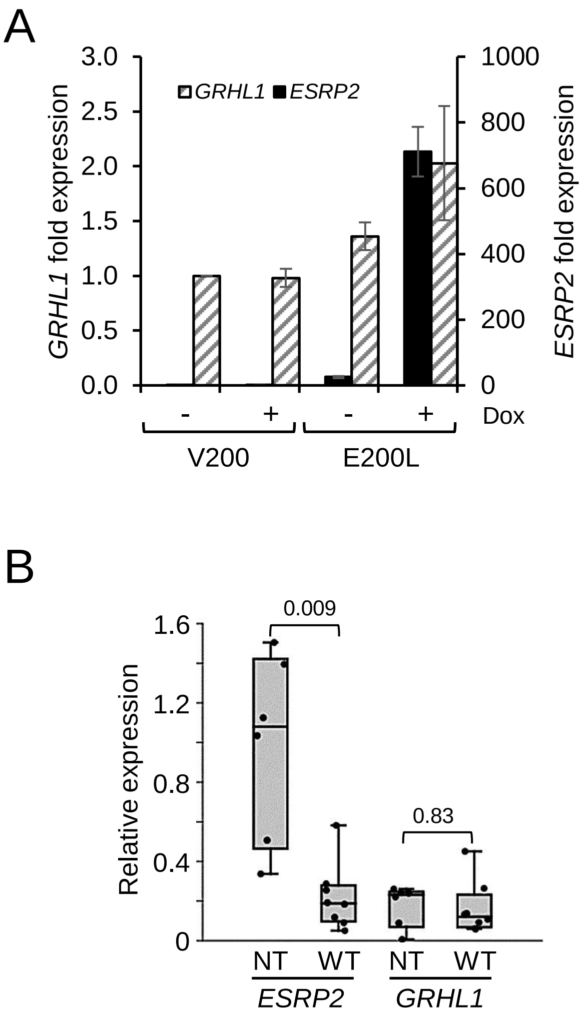

Figure S14: *GRHL1* RNA expression

A: QPCR of *ESRP2* and *GRHL1* RNA expression, normalized to endogenous levels of *TBP*, in Dox-induced and uninduced V200 and E200L cells. Expression shown as means ± SD of n=3, relative to uninduced V200 cells.

B: Dot-boxplot QPCR of *ESRP2* and *GRHL1* RNA expression, normalized to endogenous levels of *TBP*. NT n=6 (3 NK and 3 FK) and WT n=8, p values from t test.

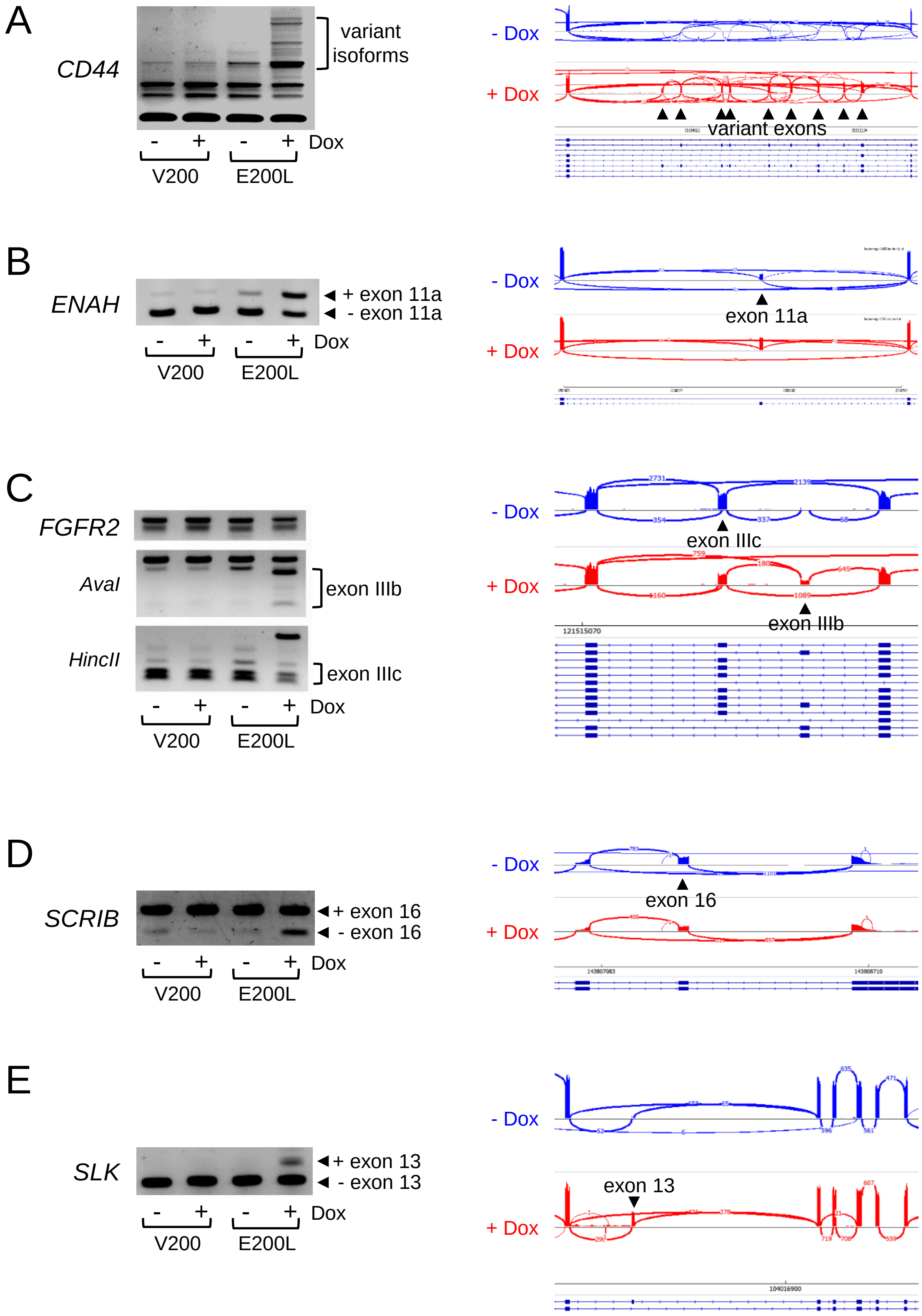

Figure S15: Successfully validated putative ESRP2 target genes
Left-hand panels: Target genes were amplified by RT-PCR for alternatively spliced exons (see supplementary table S1 for primers). *FGFR2* exons were detected by restriction enzyme cleavage (Warzecha et al (2009) Mol. Cell 33(5): 591-601) . Right-hand panels: Sashimi plots of RNA-seq data from E500L cells uninduced (-Dox) or induced to express ESRP2 (+Dox).

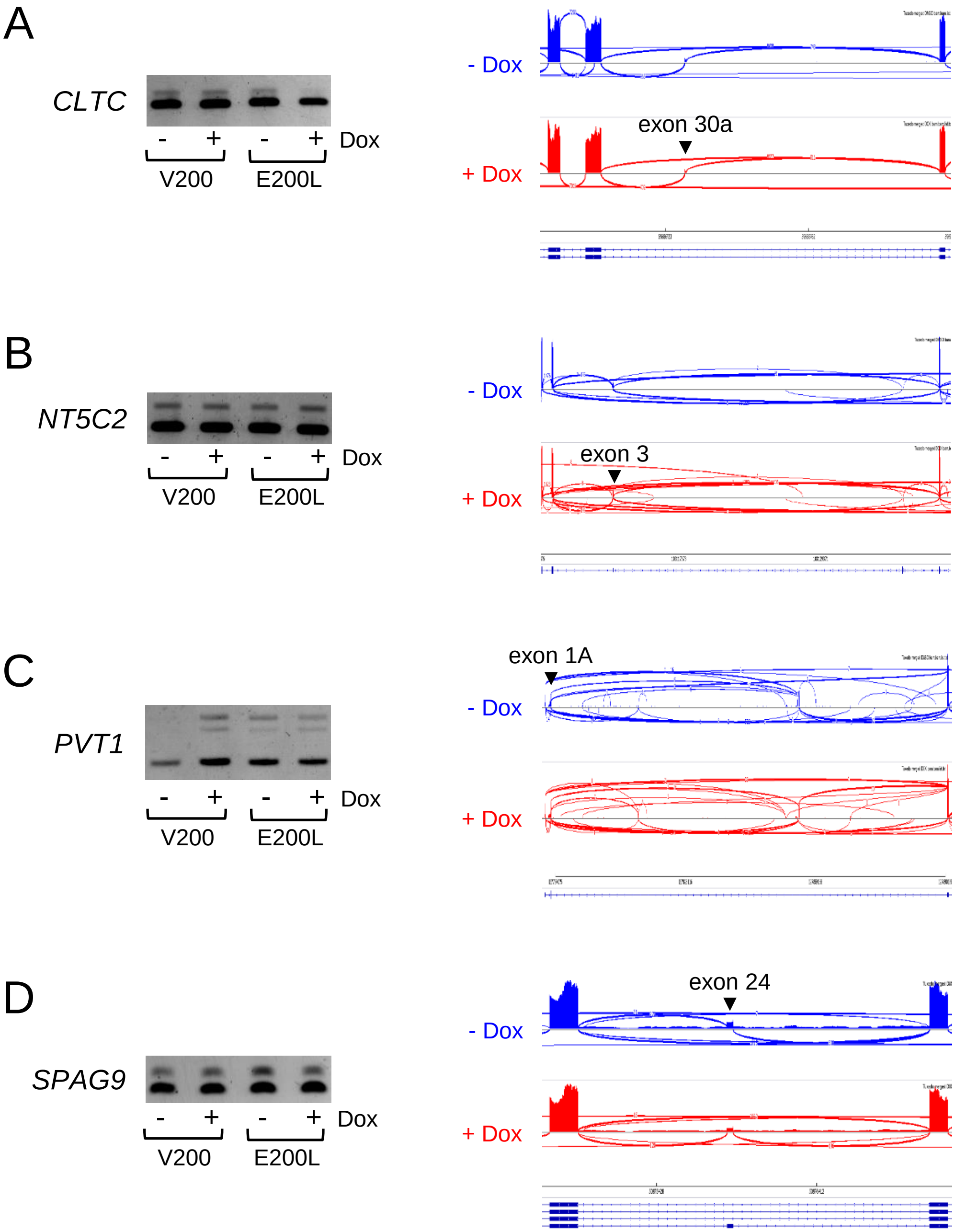

Figure S16: Unsuccessfully validated putative ESRP2 target genes
Left-hand panels: Target genes were amplified by RT-PCR for alternatively spliced exons (see supplementary table S1 for primers).
Right-hand panels: Sashimi plots of RNA-seq data from E500L cells uninduced (-Dox) or induced to express ESRP2 (+Dox).

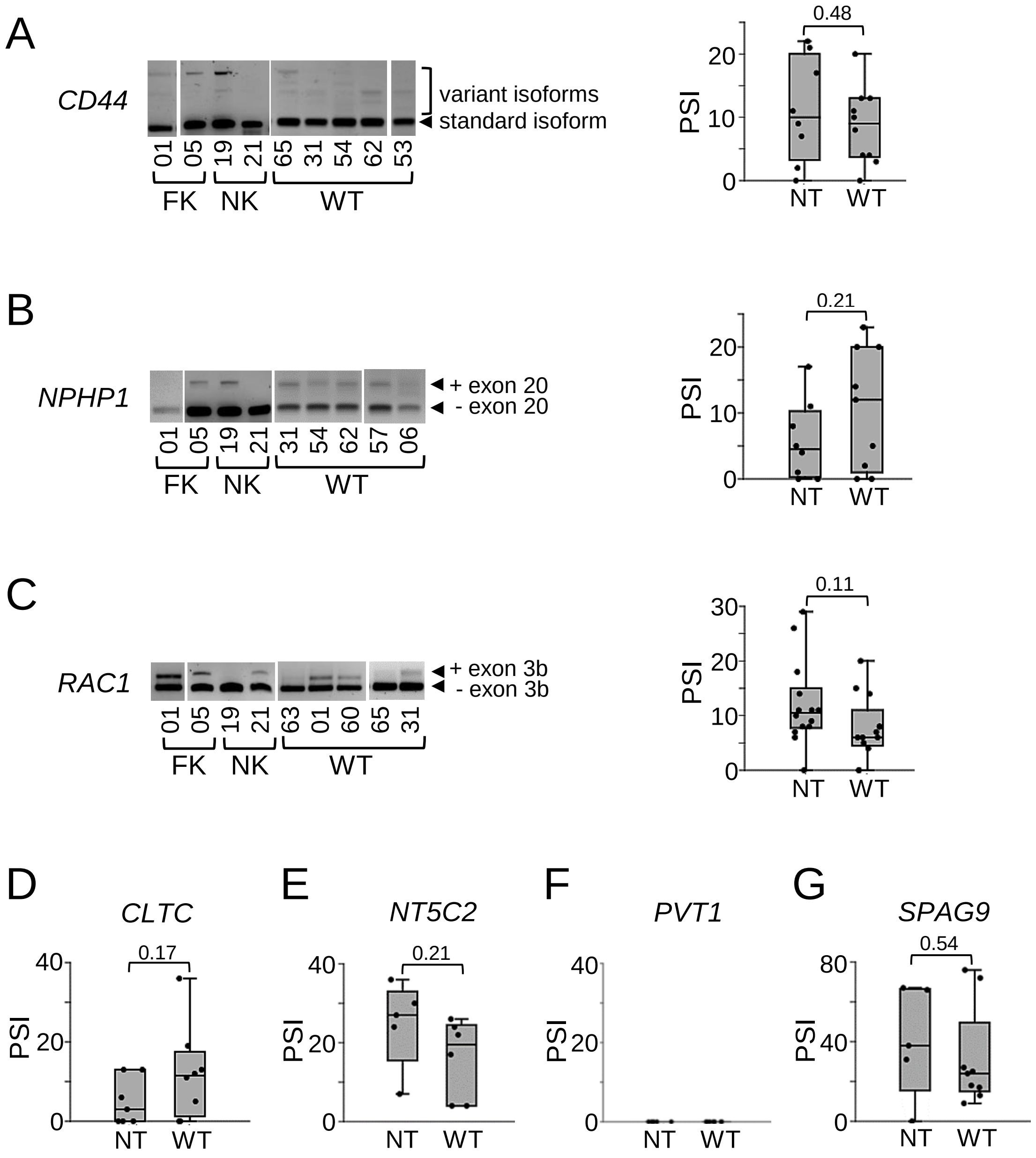

Figure S17: Alternative splicing of putative ESRP2 target genes in normal kidney and Wilms tumour

A to C: Left-hand panels: Target genes were amplified by RT-PCR for alternatively spliced exons (see supplementary table S1 for primers). Right-hand panels: Dot-Boxplots showing percent splice inclusion (PSI) in normal tissues (NT; NK and FK) and WT. p values from t test. **D to G:** Dot-boxplots showing percent splice inclusion (PSI) in normal tissues (NT; NK and FK) and WT. p values from t test.
